# Unveiling the Genome-Wide Consequences of Range Expansion and Mating System Transitions in *Primula vulgaris*

**DOI:** 10.1093/gbe/evae208

**Published:** 2024-09-28

**Authors:** Emiliano Mora-Carrera, Rebecca L Stubbs, Giacomo Potente, Narjes Yousefi, Simon Aeschbacher, Barbara Keller, Rimjhim Roy Choudhury, Ferhat Celep, Judita Kochjarová, Jurriaan M de Vos, Peter Szövényi, Elena Conti

**Affiliations:** Department of Systematic and Evolutionary Botany, University of Zurich, Zurich, Switzerland; Department of Systematic and Evolutionary Botany, University of Zurich, Zurich, Switzerland; Department of Systematic and Evolutionary Botany, University of Zurich, Zurich, Switzerland; Department of Systematic and Evolutionary Botany, University of Zurich, Zurich, Switzerland; Department of Evolutionary Biology and Environmental Studies, University of Zurich, Zurich, Switzerland; Department of Systematic and Evolutionary Botany, University of Zurich, Zurich, Switzerland; Department of Systematic and Evolutionary Botany, University of Zurich, Zurich, Switzerland; Department of Biology, Faculty of Engineering and Natural Sciences, Kırıkkale University, Kırıkkale, Turkey; Department of Phytology, Faculty of Forestry, Technical University in Zvolen, Zvolen, Slovak Republic; Department of Environmental Sciences—Botany, University of Basel, Basel, Switzerland; Department of Systematic and Evolutionary Botany, University of Zurich, Zurich, Switzerland; Department of Systematic and Evolutionary Botany, University of Zurich, Zurich, Switzerland

**Keywords:** range expansions, genomic selfing syndrome, mating system transitions, heterostyly, homostyly, quaternary glaciations

## Abstract

Genetic diversity is heterogeneously distributed among populations of the same species, due to the joint effects of multiple demographic processes, including range contractions and expansions, and mating systems shifts. Here, we ask how both processes shape genomic diversity in space and time in the classical *Primula vulgaris* model. This perennial herb originated in the Caucasus region and was hypothesized to have expanded westward following glacial retreat in the Quaternary. Moreover, this species is a long-standing model for mating system transitions, exemplified by shifts from heterostyly to homostyly. Leveraging a high-quality reference genome of the closely related *Primula veris* and whole-genome resequencing data from both heterostylous and homostylous individuals from populations encompassing a wide distribution of *P. vulgaris*, we reconstructed the demographic history of *P. vulgaris*. Results are compatible with the previously proposed hypothesis of range expansion from the Caucasus region approximately 79,000 years ago and suggest later shifts to homostyly following rather than preceding postglacial colonization of England. Furthermore, in accordance with population genetic theoretical predictions, both processes are associated with reduced genetic diversity, increased linkage disequilibrium, and reduced efficacy of purifying selection. A novel result concerns the contrasting effects of range expansion versus shift to homostyly on transposable elements, for the former, process is associated with changes in transposable element genomic content, while the latter is not. Jointly, our results elucidate how the interactions among range expansion, transitions to selfing, and Quaternary climatic oscillations shape plant evolution.

**Significance**

Demographic events such as range expansions and transitions from outcrossing to selfing are among the major determinants of the uneven distribution of genetic diversity among populations. By using whole-genome resequencing data, we demonstrate that an east-to-west range expansion followed by a recent mating system transition in the common Primrose (*Primula vulgaris*) is associated with a decrease in genetic diversity, an increase in linkage disequilibrium, and a reduction in the efficacy of purifying selection. Overall, our study provides important insights into the genomic consequences of Quaternary range expansions and reproductive strategies.

## Introduction

Understanding the processes that govern the distribution of genetic diversity in space and time is a fundamental aim of evolutionary biology. A prevalent pattern is that genetic variation within species is not uniformly distributed across populations (Ellegren and Galtier 2016). Differences in genetic diversity among populations can be driven by multiple processes, including past demographic changes and mating system transitions (Hewitt 2004; Cutter 2019). For instance, population expansions and contractions in response to climatic fluctuations can cause changes in genetic diversity over time and space. A prominent example of this is the impact of the Quaternary glacial and interglacial periods on the distribution of genetic diversity in numerous plant species across different biogeographic regions (Hewitt 2004). In addition to past demographic history, transitions from outcrossing to selfing play a crucial role in determining the partitioning of genetic diversity within and among populations (Cutter 2019). It has been shown that, in the long term, this transition increases the probability of extinction at the species level (Goldberg et al. 2010; de Vos et al. 2014), thus determining macroevolutionary patterns.

The effects of range expansions and mating system transitions on genetic diversity are mediated by changes in effective population size (*N_e_*; Ellegren and Galtier 2016). Specifically, range expansions are accompanied by an increase of homozygosity reflecting a gradual decrease in *N_e_* from the core population to the expanding range front (Peischl and Excoffier 2015). Successive contractions of *N_e_* due to repeated founder events at the range edge do not only lead to a progressive reduction of genetic diversity but also an increase of linkage disequilibrium (LD) from the ancestral source population to the most recently established populations (Excoffier et al. 2009; Lucek and Willi 2021). Moreover, the reduction in *N_e_* toward range edges increases the impact of genetic drift and reduces the efficacy of purifying selection (Charlesworth 2009). Thus, recently established populations that went through a genetic bottleneck are more likely to accumulate slightly deleterious mutations, a phenomenon known as “expansion load” (Peischl et al. 2013). Similarly to range expansions, the transition from outcrossing to selfing is associated with a decline in *N_e_* and increased homozygosity, resulting in a sharp decrease in genome-wide diversity, an increase in LD, reduced efficacy of purifying selection, and increased purging of recessive deleterious mutations (Wright et al. 2008; Arunkumar et al. 2015; Mattila et al. 2020; Lucek and Willi 2021; Wang et al. 2021). These genomic consequences of mating system transitions are part of the so-called genomic selfing syndrome (Wright et al. 2008; Cutter 2019).

The decrease of *N_e_* following range expansions and transitions to selfing can also affect the content of transposable elements (TEs) in the genome. Specifically, a reduced efficiency of purifying selection resulting from decreased *N_e_* is predicted to cause an increase in TEs that would otherwise be eliminated by natural selection (Rouzic and Deceliere 2005). Therefore, a greater accumulation of TEs at the margins than at the center of a species’ distributional range and in selfing than in outcrossing populations is expected. Conversely, high levels of homozygosity, as observed during both range expansions and mating system transitions, may facilitate the purging of recessive deleterious TEs that are concealed from selection in a heterozygous state (Wright and Schoen 2000; Morgan 2001). Furthermore, in populations with small *N_e_*, newly arisen TEs are more likely to be lost due to increased genetic drift (Boutin et al. 2012). Consequently, a lower genomic TE content at the distributional range margins and in selfing populations than in the center of a species’ distributional range and/or in outcrossing populations can also be expected. Evidence for both an increase and a decrease of genomic TE content following reductions of *N_e_* due to range expansions and mating system transitions has been reported in Brassicaceae (Bunchev and Willi 2018; Mattila et al. 2020; Jiang et al. 2022). Despite the importance of TEs in shaping genome evolution, there have been limited efforts to investigate the consequences of range expansions and mating system transitions on genomic TE content in non-Brassicaceae model species.

The common primrose (*Primula vulgaris* Huds.) is an ideal model to study the genomic consequences of range expansions and mating system transitions owing to the wealth of previous knowledge on ecology and evolutionary biology of this species (Richards 2003; Jacquemyn et al. 2009). Previous phylogenomic work indicated that *P. vulgaris* and the closely related *Primula veris* (L.) Hill and *Primula elatior* (L.) Hill originated in the Caucasus and expanded from east to west (Stubbs et al. 2023). The age of *Primula* sect. *Primula*, which includes *P. vulgaris* and *P. veris*, was estimated at ca. 1.53–3.27 million years (de Vos et al. 2014). Additionally, it was proposed that the European populations of *P. vulgaris* originated more recently, after postglacial expansion during the Quaternary (Volkova et al. 2020). Further phylogeographic work corroborated the recent westward range expansion of *P. vulgaris* (Triest et al. 2024). However, whether the range expansion is associated with a reduction of genome-wide diversity, increased LD, decreased efficacy of purifying selection, and differences in the accumulation and genomic content of TEs in the genome has not been investigated. The recently developed chromosome-scale, highly complete genome assembly of *P. veris* (Potente et al. 2022) now enables the testing of the above predictions about the timing and genomic consequences of range expansion in *P. vulgaris*.

**Fig. 1.**
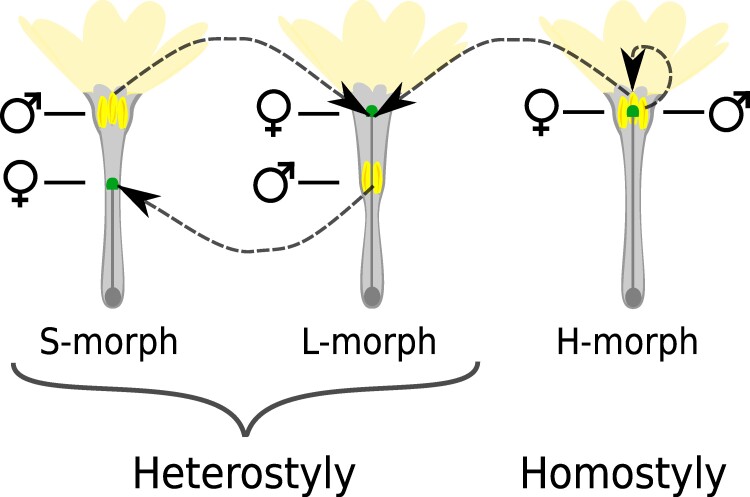
Representation of heterostyly and homostyly. In *Primula*, heterostyly is characterized by two distinct floral morphs (distyly) that differ in the position of male (i.e. anthers) and female (i.e. stigma) sexual organs. In the S-morph, anthers (yellow) are positioned above the stigma (green), whereas the L-morph has the reciprocal arrangement. In *Primula*, distyly is often accompanied by a system of intramorph incompatibility preventing self-fertilization. Homostyly (H-morph) is characterized by proximity of sexual organs within the flower and loss of self-incompatibility. Arrows indicate predominant directions of successful pollination.

*Primula vulgaris* represents a long-standing system for the study of mating system transitions because, in this species, homostyly, which enables selfing, has repeatedly evolved from heterostyly, which promotes outcrossing (Crosby 1949; Li et al. 2016; Mora-Carrera et al. 2023). The most common type of heterostyly is distyly, characterized by the cooccurrence of two types of usually self-incompatible individuals distinguished by complementary floral morphs: the short-styled morph (S-morph) with anthers above the stigma and the long-styled morph (L-morph) with the reciprocal arrangement of sexual organs (Fig. 1). This reciprocal positioning of sexual organs between floral morphs promotes pollen export from L- to S-individuals and vice versa (Keller et al. 2014). Conversely, homostyly denotes self-compatible floral morphs (H-morphs) characterized by absent or markedly reduced spatial separation between anthers and stigma. The proximity of sexual organs in homostylous flowers favors self-pollen deposition, facilitating self-fertilization (Fig. 1). In *Primula*, homostyles originate via mutations in a gene called *CYP^T^* located in a hemizygous region known as the heterostyly *S*-locus supergene that is present in S-morph individuals but absent from L-morph individuals; it has been shown that *CYP^T^* determines short stigmas in S-morphs and that mutations disrupting its expression cause style elongation and the ensuing shift to homostyly (Huu et al. 2016; Li et al. 2016; Mora-Carrera et al. 2023).

Evidence of higher selfing rates in homostyles has been reported in multiple species across various families (Crosby 1959; Belaoussoff and Shore 1995; Johnston and Schoen 1996; Yuan et al. 2017; Zhou et al. 2017; Zhong et al. 2019; Mora-Carrera et al. 2023). Furthermore, previous studies revealed higher homozygosity in homostylous than heterostylous taxa (Piper et al. 1986; Ness et al. 2010; Yuan et al. 2017; Zhou et al. 2017; Zhong et al. 2019). In *P. vulgaris*, an increase of homozygosity associated with an increase in the frequency of homostyles within populations was detected using microsatellites (Mora-Carrera et al. 2023). However, despite the long-standing value of *P. vulgaris* as a model for studying transitions to selfing, whether features of the genomic selfing syndrome such as reduced genome-wide diversity, increased LD, and less efficient purifying selection are present in this species has never been investigated. Indeed, the genomic resources necessary to address these questions have only recently become available.

Finally, *P. vulgaris* represents an ideal system to disentangle the tempo and mode of the transitions to selfing in relation to range expansion. In *P. vulgaris*, heterostylous populations are widely distributed from the Caucasus to mainland Europe, including most Mediterranean islands and all the British Isles (Jacquemyn et al. 2009). In contrast, homostylous individuals have only been reported from populations in Somerset, England (Crosby 1940, 1949; Curtis and Curtis 1985) and from one population in the Netherlands (Barmentlo et al. 2018). It was suggested that homostyly could have originated outside England and subsequently colonized Somerset (Charlesworth 2023), which would imply that the shift to homostyly predated the colonization of the British Isles. Alternatively, homostyles may have originated within England (Mora-Carrera et al. 2023), in which case colonization would have preceded the shift to homostyly. The combination of phylogenetic evidence with the genomic signatures of *N_e_* reductions and the knowledge that homostyles originate via mutations in the hemizygous *S*-locus of S-morph individuals (Huu et al. 2016; Li et al. 2016; Mora-Carrera et al. 2023) allows us to test the following parsimonious predictions with respect to the relative order of colonization and the shift to homostyly. In the first scenario, if homostyly evolved locally in England after the colonization of the island, we expect English homostyles to be more closely related to English than European heterostyles. Moreover, a bottleneck associated with the shift to homostyly should be preceded by a bottleneck associated with the colonization of England. Alternatively, if homostyly preceded the colonization of England, we expect English homostyles to be more closely related to European than English heterostyles. In addition, the bottleneck associated with the colonization of England should be more recent than the bottleneck associated with the shift to homostyly. Until now, the relative order of the colonization of England and the shift to homostyly in *P. vulgaris* has remained unknown. Furthermore, whether the English populations of *P. vulgaris* stem from a single or multiple island colonizations, i.e. whether English populations are monophyletic or not, remains unknown. The missing knowledge precludes a deeper understanding of how the interactions among range expansion, transitions to selfing, and Quaternary climatic oscillations shape plant evolution.

Here, we analyzed whole-genome resequencing data from 105 samples encompassing the distribution of *P. vulgaris* from Turkey to England to study the genomic consequences of range expansions and the shift to homostyly. Specifically, we addressed the following questions: (i) Do phylogenetic and population genetic analyses support the out-of-Caucasus range expansion of *P. vulgaris* and the monophyly of its English populations? (2) Are the out-of-Caucasus expansion, colonization of England, and shift/s to homostyly in *P. vulgaris* associated with reductions of *N_e_*? If so, when did these genetic contractions (bottlenecks) occur? (iii) Do range expansion and shift/s from heterostyly to homostyly in *P. vulgaris* lead to a decrease of genome-wide diversity, increase of LD, reduction in the efficacy of purifying selection, and differences of genomic TE content? By answering the above questions, the present study generates new knowledge on how different processes shape the heterogeneous partitioning of genetic diversity within species.

## Results

**Fig. 2.**
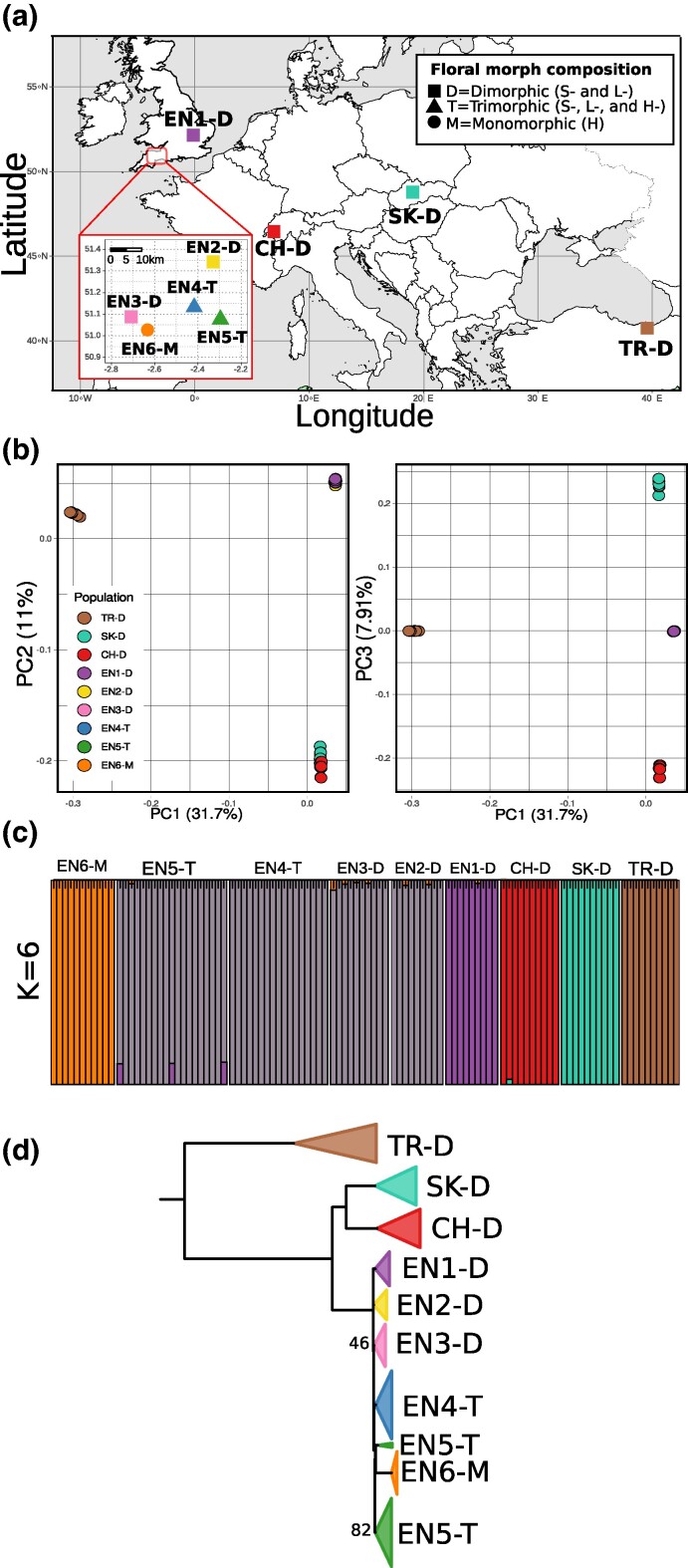
Overview of the populations of *P. vulgaris* sampled for this study and respective population genetic and phylogenetic results inferred from analyses of whole genome resequencing data. a) Geographic location and floral morph composition of the nine populations used in this study. The expanded inset includes dimorphic, trimorphic, and monomorphic populations from Somerset, England. b) 2D plot of the first three PCs (PC1 vs PC2 and PC1 vs PC3) of the PCA and respective percentage of explained genetic variation. c) Results of the ADMIXTURE analysis of the sampled populations, where *K* represents the number of genetic ancestry clusters (indicated with different colors) used for the analyses (only results for *K* = 6 are shown). d) ML phylogeny of all 105 *P. vulgaris* samples based on 1,532,562 genome-wide SNPs. Terminal branches of individuals from the same population were collapsed. All but two (labeled) internal branches received bootstrap support (BS) > 95%. Outgroups are not shown. CH, Switzerland; EN, England; SK, Slovakia; TR, Turkey.

To test the consequences of range expansion and mating system transition, we sampled 105 individuals of *P. vulgaris* from six dimorphic, heterostylous populations (comprising only S- and L-morph individuals) in continental Europe (Turkey: TR-D; Slovakia: SK-D; Switzerland: CH-D) and England (EN1-D, EN2-D, and EN3-D); two trimorphic populations (comprising S-, L-, and H-morph individuals) in England (EN4-T and EN5-T); and one monomorphic population (comprising exclusively H-morph individuals) from England (EN6-M; Fig. 2a; Table 1). We note that population TR-D stems from a region in northeastern Turkey that is adjacent to the core of the Caucasus region.

**Table 1:** Summary of sampled individuals and population genetic statistics from nine populations of *P. vulgaris*

Population	Sampled individuals per floral morph			S_syn_	Θw_-syn_	π_syn_	π_nonsyn_/π_syn_	*D*
	S	L	H					
TR-D	5	5	…	72,295	0.0045	0.0033	0.4265	−0.9261
SK-D	5	5	…	23,992	0.0016	0.0014	0.4615	−0.2225
CH-D	5	5	…	36,226	0.0022	0.0018	0.4432	−0.5367
EN1-D	5	4	…	18,261	0.0012	0.0011	0.4435	−0.2595
EN2-D	4	5	…	17,430	0.0012	0.0011	0.4735	−0.0399
EN3-D	5	5	…	16,193	0.0011	0.0010	0.4689	−0.0158
EN4-T	3	4	…	11,778	0.0008	0.0008	0.4744	0.0400
	…	…	10	13,179	0.0008	0.0008	0.4732	0.0371
EN5-T	5	4	…	12,852	0.0008	0.0008	0.5093	−0.1436
	…	…	10	12,235	0.0008	0.0007	0.4953	0.0078
EN6-M	…	…	11	2536	0.0001	0.0001	0.5370	0.5412

Calculated diversity estimates include the number of segregating sites (S), Watterson's theta (θ_w_), pairwise nucleotide diversity (π), the ratio of π at nonsynonymous (non-syn; 0-fold sites) to synonymous (syn; 4-fold sites) sites, and Tajima's *D* (*D*) at synonymous sites. All estimates except S_syn_ are the genome-wide average over 50,000 nonoverlapping windows. In the trimorphic populations (EN4-T and EN5-T), population genetic estimates were calculated separately for S- and L-individuals (heterostyles) combined vs. H-individuals (homostyles). Population codes are as in Fig. 1.

On average, 96.76% ± 1.39 (mean ± SD) of the sequencing reads per individual of *P. vulgaris* mapped to the reference genome of *P. veris*. Sequencing depth ranged from 13.02 to 28× (mean ± SD; 18.9 ± 3.08). The final set of single nucleotide polymorphisms (SNPs) after filtering included 96,683,395 biallelic SNPs.

### Genetic Structure and Phylogeny of *P. vulgaris*

To explore the genetic structure of our populations, we conducted a principal component analysis (PCA). The PCA showed that principal components (PCs) 1, 2, and 3 jointly accounted for 50.59% of the genetic variation (31.71%, 10.97%, and 7.91%, respectively). The 2D plot of these three PCs indicated that each continental population (TR-D, SK-D, and CH-D) forms a separate cluster, whereas all English populations are grouped in a single cluster (Fig. 2b). To further explore population structure and signatures of genetic admixture, we ran ADMIXTURE v1.3 (Alexander et al. 2009). The ADMIXTURE analysis showed a clear genetic differentiation between the continental populations and all English populations (K = 5; Fig. 2c). Under the best-fitting model of the ADMIXTURE analysis (K = 6; supplementary table S1, Supplementary Material online), all English populations but the homostylous monomorphic population (EN6-M) were found to form a single genetic group (Fig. 2c). Finally, the maximum likelihood (ML) genome-wide phylogeny (iq-tree model [TVMe + ASC + R2]) is well resolved and shows high bootstrap support (>95%) on most internal nodes (Fig. 2d). In accordance with the PCA and ADMIXTURE results, the ML phylogeny inferred with IQ-tree v2.1.2 (Minh et al. 2020) revealed that TR-D is sister to a clade comprising all remaining populations and that the SK-D and CH-D populations form a clade sister to the clade comprising all English populations. Furthermore, all English populations were found to be monophyletic, but relationships among them remained unresolved (Fig. 2d). The monomorphic homostylous population, EN6-M, was found to be embedded in the clade comprising all English heterostylous populations, implying that the shift to homostyly occurred after the colonization of the island.

### Demographic History of *P. vulgaris*

**Fig. 3.**
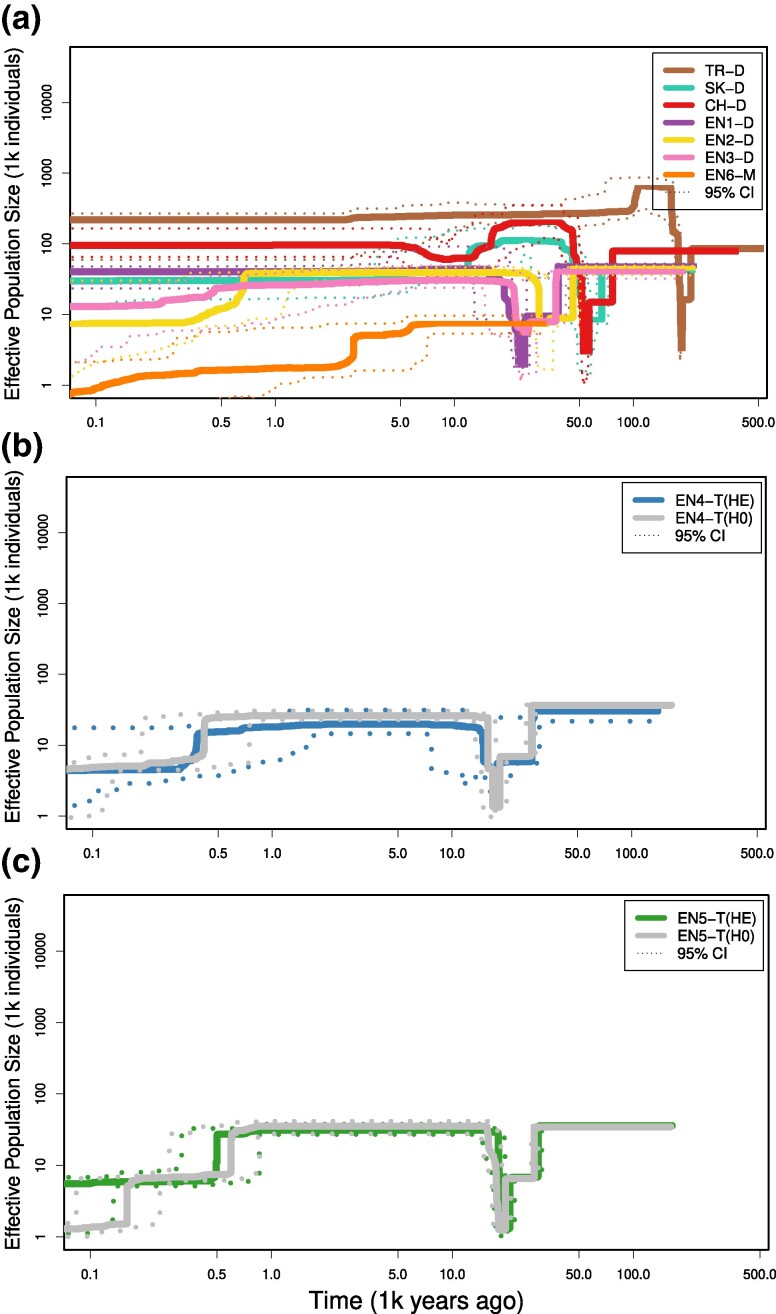
Single-population trajectories of effective population size through time for nine populations of *P. vulgaris*. a) Stairway plots for six dimorphic heterostylous (TR-D, SK-D, CH-D, EN1-D, EN2-D, and EN3-D) populations and one monomorphic homostylous (EN6-M) population inferred with Starwaiplot2 (see Materials and Methods). The *x* axis represents time in thousands of years from the present (left) to the past (right) on a logarithmic scale, while the *y* axis represents the effective population size (*P. vulgaris*); 95% confidence intervals (CI) for *P. vulgaris* are represented with dotted lines. Populations are represented in different colors, as in Fig. 1. b, c) Stairway plots of the trimorphic populations (EN4-T and EN5-T, respectively). In each trimorphic population, individuals were divided into heterostyles (HE; i.e. S- and L-morph individuals) and homostyles (HO; i.e. H-morph individuals shown in gray). For all analyses, a mutation rate of 1.23 × 10^−8^ per site per generation and a 2-year generation time were assumed (see Materials and Methods).

We inferred single-population demographic histories using Stairway Plot v2 (Liu and Fu, 2020). The stairway plots suggested that the Turkish population (TR-D) experienced a genetic bottleneck ca. 178,000 to 210,000 years before present (ybp) and has maintained a stable *N_e_* of ca. 200,000 over the last ca. 100,000 years until present (Fig. 3a). Both the Slovakian (SK-D) and Swiss (CH-D) populations experienced a potentially shared genetic bottleneck ca. 40,000 to 67,000 ybp and subsequently maintained stable *N_e_* of ca. 40,000 and 100,000 over the last ca. 5,000 years (Fig. 3a). All dimorphic and trimorphic populations in England seem to have experienced a more recent bottleneck ca. 15,000 to 37,000 ybp (Fig. 3a to c). Within trimorphic populations (EN4-T and EN5-T), heterostyles and homostyles showed similar trajectories of *N_e_* with a bottleneck ca. 15,000 to 37,000 ybp and an additional, recent reduction of *N_e_* ca. 400 ybp. For the English monomorphic population (EN6-M), we observed a low but stable *N_e_* ca. 7,000 to 9,000 for the last 30,000 years followed by a strong reduction of *N_e_* starting ca. 2,500 ybp and continuing to the present (Fig. 3a). We thus observed no recovery of *N_e_* in this population as of now. In general, the Turkish population has had the highest *N_e_* for the past 100,000 years (ca. 200,000) and the English monomorphic population the lowest *N_e_* for the past 2,500 years.

**Fig. 4.**
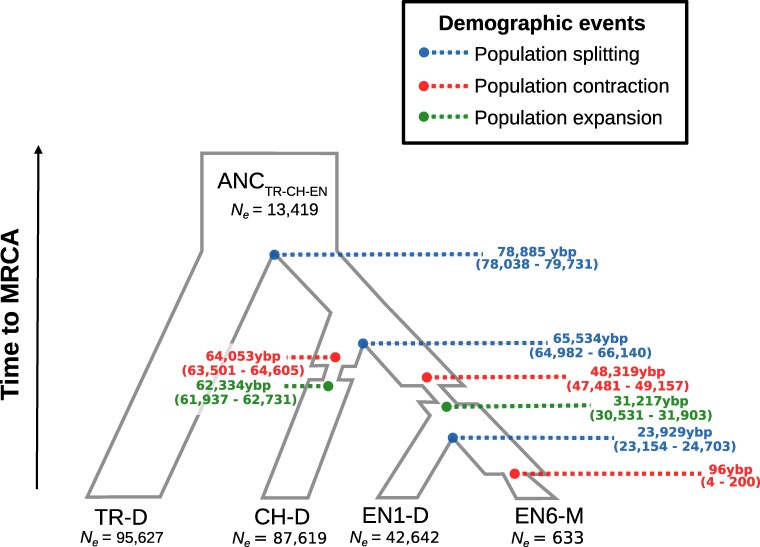
Demographic history of *P. vulgaris* according to the best-supported demographic model as inferred by *P. vulgaris*. Coalescent simulations (see Materials and Methods) were run for four populations (TR-D, CH-D, EN1-D, and EN6-M) representing the extremes of the distributional range across the sampled *P. vulgaris* populations. Branch width represents effective population size (*P. vulgaris*) of each population. Blue dots indicate splits between populations, whereas red and green dots represent population contraction or expansion, respectively; ANC_TR-CH-EN_ represents the Most Recent Common Ancestor (MRCA) of all *P. vulgaris* populations. The timing of each demographic expansion or contraction is expressed as years before present; 95% confidence intervals (CI) for the timing of demographic events are indicated in parenthesis.

Given that the single-population stairway plots above use a model-free method to reconstruct demographic history, we incorporated these presumed bottlenecks to test six specific demographic scenarios (supplementary fig. S1, Supplementary Material online) and infer demographic events under these models based on the multidimensional site frequency spectrum (SFS) using *fastsimcoal2*. The best-fitting demographic model selected via Akaike information criterion (AIC) was Model 5 (AIC_Model-4_ = 396,297; supplementary figs. S1 and S2, Supplementary Material online). Under Model 5, the split between the Turkish and European populations of *P. vulgaris* was estimated at ca. 79,000 ybp (Fig. 4). This estimate implies that the split occurred after the genetic bottleneck inferred for TR-D using the stairway plots (Fig. 3a), which would also be compatible with the reduced *N_e_* in the most recent common ancestral population of all four populations (ANC_TR-CH-EN_; *N_e_* = 13,419) when compared to the current *N_e_* of TR-D (*N_e_* = 95,627). According to the model, the split between the Swiss population and the English population was dated to ca. 65,500 ybp. This split was quickly followed by a population contraction ca. 64,000 ybp and a later population recovery ca. 62,300 ybp in the CH-D and an independent population contraction ca. 48,000 ybp and a later population recovery ca. 31,000 ybp in the lineage that gave rise to the English populations. The split between the easternmost English population EN1-D and the monomorphic population EN6-M was inferred to have occurred ca. 24,000 ybp and was followed by a very recent population genetic contraction in EN6-M ca. 100 ybp.

### Genomic Consequences of Range Expansion and Shift to Homostyly

#### Genome-Wide Diversity

**Fig. 5.**
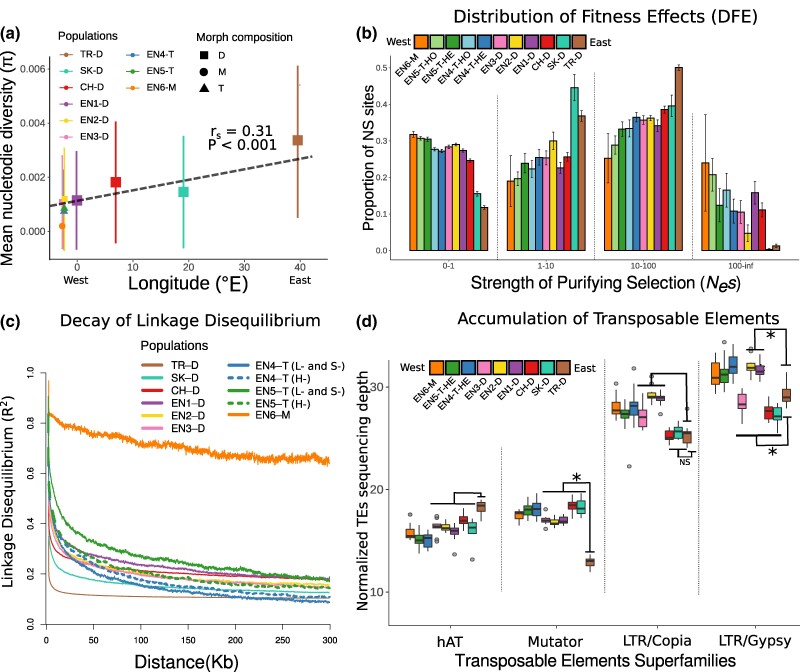
Genomic consequences of range expansion and the transition from heterostyly to homostyly in *P. vulgaris.* a) Correlation plot showing the relationship between mean nucleotide diversity (π) at synonymous sites (4-fold) and longitude coordinates (°E). Significance of the correlation was estimated for Spearman's rank correlation coefficient (*r*s). b) Shifts in the DFE within populations. From left to right, the *x* axis shows four bins with increasing strength of purifying selection (*P. vulgaris*) as follows: nearly neutral sites (*P. vulgaris*, from 0 to 1); weakly selected sites (*P. vulgaris*, from 1 to 10); strongly selected sites (*P. vulgaris*, from 10 to 100); and very strongly selected sites (*P. vulgaris*, from 100 to infinity). The *y* axis shows the proportion of nonsynonymous (NS; 0-fold degenerate) sites in each bin. c) Decay of LD (measured as *r*^2^; *y* axis) over physical distance along the genome in kilobase (*x* axis) in each population. d) Prevalence of different DNA (*P. vulgaris.* and *P. vulgaris.*) and RNA (*P. vulgaris.* and *P. vulgaris.*) superfamilies of TEs in each population. The prevalence of TEs in the genome was estimated as the sequencing depth of each TE family normalized by the sequencing depth of each sample (see Materials and Methods). Significance was calculated using the pairwise Wilcoxon test with Bonferroni correction establishing TR-D as the intercept. Significant differences (*P* < 0.05) are marked by an asterisk (*).

To explore genome-wide signatures of range expansion and shift to homostyly, we first computed population-genetic summaries of diversity and LD. We detected a significantly positive correlation between longitude and estimates of genome-wide nucleotide diversity (π_S_) and Watterson's θ (θ_W-S_) (*r_s_* = 0.31, *P* < 0.001 for π_S_, Fig. 5a; and *r_s_* = 0.43, *P* < 0.001 for θ_W-S_; Table 1). This result supports the predicted reduction of genome-wide diversity concomitant with range expansion from the southeast (Turkey) to the northwest (England). Among English populations, the Kruskal–Wallis test and its subsequent pairwise Wilcoxon-rank tests revealed that, as predicted after a shift to homostyly, π_S_ and θ_W-S_ were significantly lower for EN6-M than for all dimorphic and trimorphic populations in England (*χ*^2^*_df_*_= 7_ = 5285.8, *P* < 0.001 for π_S_, and *χ*^2^*_df_*_= 7_ =5623.5, *P* < 0.001 for θ_W-S_; Table 1). Furthermore, genomic diversity was significantly higher in dimorphic (EN1-D, EN2-D, and EN3-D) than trimorphic populations (EN4-T and EN5-T; Table 1). However, within trimorphic populations, heterostyles and homostyles maintained similar levels of genetic diversity (Table 1 and supplementary table S2, Supplementary Material online).

In agreement with the serial bottlenecks following range expansion (see above), we observed an east-to-west increase of Tajima's *D*, with the lowest value found in TR-D (*D* = −0.926; Table 1). Within England, the homostylous monomorphic population (EN6-M) had the highest Tajima's *D* value (*D* = 0.541), indicating a loss of low-frequency alleles and an increase of intermediate and high-frequency alleles, as expected after a recent genetic contraction.

Finally, the lowest level of LD was detected in the Turkish population, with the sharpest increase in the monomorphic English population EN6-M. This latter population had a ca. 4-fold increase in LD relative to the dimorphic populations in England (EN1-D, EN2-D, and EN3-D), a result that can be attributed to the transition to homostyly and full monomorphy in EN6-M. In dimorphic populations, levels of LD increased from east to west, confirming theoretical population genetic predictions of range expansion. However, interpopulation differences of LD associated with range expansion were not pronounced, suggesting that bottlenecks were not very severe and/or insufficient time has passed to reach equilibrium after such bottlenecks. Concerning the trimorphic populations, levels of LD decay were similar in heterostyles and homostyles of EN4-T, as were the levels of genetic diversity (Fig. 5c). We note that LD in EN5-T was higher in the heterostyles than in the homostyles. However, we cannot propose an explanation that can be tested with our current data.

#### Strength of Purifying Selection

To test population-genomic predictions suggesting a relaxation of purifying selection in response to higher rates of selfing after the shift to homostyly, we explored the ratio of synonymous versus nonsynonymous nucleotide diversity (π_N_/π_S_) as well as the distribution of fitness effects (DFE). As expected after a range expansion from east to west, we observed a weak but statistically significant negative correlation between π_N_/π_S_ and longitude (*r_s_* = −0.08, *P* < 0.001; supplementary fig. S3, Supplementary Material online). Moreover, our DFE analysis showed a strongly significant positive correlation between the proportion of nearly neutral nonsynonymous sites (0 ≤ *N_e_s* < 1) and longitude (*r_s_* = −0.93, *P* < 0.001; Fig. 5b and supplementary fig. S4a, Supplementary Material online). Both results thus suggest an east-to-west decrease in the efficacy of purifying selection, i.e. a higher proportion of potentially deleterious, but effectively neutral, nonsynonymous mutations segregating in the smaller western populations compared to the larger eastern populations. In contrast to the nearly neutral nonsynonymous sites, we found an east-to-west reduction in the proportion of nonsynonymous mutations putatively under weak (1 ≤ *N_e_s* < 10; *r_s_* = 0.58, *P* < 0.001; supplementary fig. S4b, Supplementary Material online) and strong (10 ≤ *N_e_s* < 100; *r_s_* = 0.74, *P* < 0.001; supplementary fig. S4c, Supplementary Material online) purifying selection, implying that potentially deleterious nonsynonymous mutations are purged more efficiently in eastern populations than in western populations. Finally, we found a negative correlation between longitude and the proportion of nonsynonymous mutations putatively under very strong purifying selection (100 ≤ *N_e_s <* ∞; *r_s_* = −0.55, *P* < 0.001; supplementary fig. S4d, Supplementary Material online), implying that very strongly deleterious mutations very rarely get fixed in the larger eastern populations.

As expected after a transition from outcrossing to selfing, hence, a decrease in effective population size and a putatively increased rate of purging, the DFE in the monomorphic homostylous population EN6-M in England showed a significant excess in the proportion of nearly neutral mutations relative to all other populations (0 < *N_e_s* ≤ 1; *χ*^2^*_df_*_= 7_ = 711.78, *P* < 0.001; Fig. 5b and supplementary fig. S4a, Supplementary Material online) and a reduction in the proportion of nonsynonymous mutations putatively under weak (1 < *N_e_s* ≤ 10; *χ*^2^*_df_*_= 7_ = 469.2, *P* < 0.001) and strong (10 < *N_e_s* ≤ 100; *χ*^2^*_df_*_= 7_ = 546.78, *P* < 0.001) purifying selection (Fig. 5b; supplementary fig. S4b and c, Supplementary Material online). Moreover, EN6-M showed a significant relative excess of mutations putatively under very strong purifying selection (100 ≤ *N_e_s <* ∞; *χ*^2^*_df_*_= 7_ = 443.74, *P* < 0.01; Fig. 5b and supplementary fig. S4d, Supplementary Material online), suggesting that mutations that are very strongly selected against are more efficiently eliminated in the homostylous population, a pattern consistent with the expected purging of recessive strongly deleterious mutations in primarily selfing populations (Arunkumar et al. 2015). Although the ratio of π_N_/π_S_ was slightly higher in EN6-M compared to the other populations (Table 1), as expected after a mating system transition, this increase was not statistically significant among the English populations (*χ*^2^*_df_*_= 7_ = 10.975, *P* = 0.13). Finally, DFE analyses in both trimorphic populations (EN4-T and EN5-T) showed higher proportions of nearly neutral nonsynonymous mutations and elevated proportions of weakly and strongly selected nonsynonymous deleterious mutations in homostyles than heterostyles, mirroring results in EN6-M (Fig. 5b). We note that, although the effect of homostyly in the 0 < *N_e_s* ≤ 1 bin of the trimorphic populations seems minor, the effects of homostyly on purifying selection within populations become clearer when considering the reduced proportion of nonsynonymous mutations putatively under weak and strong selection, respectively (i.e. *N_e_S* = 1 to 10 and 10 to 100 bins), a pattern that is consistent with the predicted reduction in the strength of purifying selection in selfers.

### Genomic Content of TEs

To quantify the potential effect of range expansion and subsequent shift to homostyly on genomic TE content, we explored genome-wide variation in TE sequencing depth as a proxy for genomic TE content. Our analyses of TE sequencing depth revealed that range expansion, but not transition to homostyly, is associated with changes in genomic TE content in *P. vulgaris*. However, different TE superfamilies showed contrasting patterns of differences in their genomic content among populations. Specifically, the genomic content of the *CACTA*, *PIF-Harbinger*, and *Tc1-Mariner* TE superfamilies did not significantly differ among populations (supplementary fig. S5 and table S3, Supplementary Material online). However, the TR-D population showed a significantly higher accumulation of *hAT* transposons compared to SK-D, CH-D, EN1-D, EN2-D, and EN3-D (Fig. 5d and supplementary fig. S5 and table S3, Supplementary Material online). In contrast, we found a lower accumulation of *Mutator* TEs in TR-D than in the remaining populations. Moreover, *Helitron* transposons were more abundant in SK-D and CH-D than in TR-D, EN1-D, EN2-D, and EN3-D (supplementary fig. S5c and table S3, Supplementary Material online. Furthermore, *LTR/Copia* had a significantly higher copy number in EN1-D, EN2-D, and EN3-D than in TR-D, SK-D, and CH-D, whereas *LTR/Gypsy* TE copy number was significantly lower in SK-D and CH-D but higher in EN1-D and EN2-D than in TR-D.

Finally, we found no significant differences in TE copy number between heterostyles from different populations in England (EN1-D, EN2-D, and EN3-D) and any of the populations where homostyles occurred (EN4-T and EN5-T), including the homostylous monomorphic population (EN6-M). Additionally, we did not recover a significant difference in TE copy number between heterostyles and homostyles within trimorphic populations (supplementary table S4, Supplementary Material online).

## Discussion

In this study, we analyzed whole-genome resequencing data from an extensive geographic sample of *P. vulgaris* to elucidate the genomic consequences of range expansion and mating system transition. Our results are consistent with a progressive east-to-west range expansion from the Caucasus region, as proposed in previous studies, starting ca. 79,000 years ago, likely driven by colonization during Quaternary interglacials. Additionally, we discovered that the colonization of the British Isles predated the shift from heterostyly to homostyly in English populations of *P. vulgaris*, supporting previous claims that homostyly evolved in England. As predicted by population genetic theory, the range expansion of *P. vulgaris* and the recent transition to homostyly are associated with a decrease of genetic diversity, an increase of LD, and a reduction in the efficacy of purifying selection, corroborating expected consequences of recent range expansions and the onset of the genomic selfing syndrome. Finally, our results indicate that the range expansion from Turkey into Europe, but not the shift to homostyly, was accompanied by changes in genomic TE content. However, differences in the change of TE content among superfamilies were detected.

### Genome-Wide Analyses Confirm an Out-of-Caucasus Range Expansion for *P. vulgaris*

Climatic oscillations due to Quaternary glacial expansions and contractions caused significant changes in the geographical distribution of various plant and animal species (Hewitt, 2004). Patterns of range contractions and expansions concomitant with glacial maxima and interglacials have been reported in many species adapted to alpine or temperate environments, including various species of *Primula* (Guggisberg et al. 2006, 2008; Theodoridis et al. 2013, 2017, 2018; Ren et al. 2017). Previous phylogeographic, phylogenomic, and ancestral range reconstruction analyses of *P. vulgaris* suggested that this species originated in the Caucasus region and subsequently expanded its distribution range from east to west during the Quaternary period (Volkova et al. 2020; Stubbs et al. 2023). Although we did not include samples from the core of the Caucasus region, our samples from northeastern Turkey stem from a population (TK-D) in an immediately adjacent region, representing a proxy for the Caucasian region proposed as the geographic origin of *P. vulgaris* in the above-mentioned studies. Our phylogenomic analysis recovered the Turkish population (TR-D), the easternmost and closest population to the Caucasus region in our study, as the sister clade of all western populations of *P. vulgaris* (Fig. 2d), providing support for the out-of-Caucasus hypothesis. Our demographic analyses indicate that the divergence between the Turkish and all northwestern populations of *P. vulgaris* occurred ca. 79,000 ybp (Fig. 4). This estimated divergence time coincides with a period of glacial contraction in the end of the Early Weichselian that occurred approximately 80,000 to 90,000 ybp (Hughes et al. 2013; Wohlfarth 2013), suggesting that *P. vulgaris* expanded its distribution northwestward as glacial retreat exposed land newly available for colonization around this time. Environmental variation across the distribution of *P. vulgaris* may influence the number of generations per year, potentially leading to a misestimation of the timing of the out-of-Caucasus range expansion. However, the age of this event inferred in our analyses overlaps with the estimated time of the range expansion from the Caucasus region of *P. vulgaris* previously estimated with microsatellites (Triest et al. 2024). Finally, as expected after range expansion originating in the Caucasus, we observed a significant decrease of genome-wide diversity and an increase of LD from east to west, with the population closest to the putative center of origin of *P. vulgaris*, namely TR-D, exhibiting the highest levels of genetic diversity and lowest levels of LD (Fig. 5a and c). These findings collectively support the notion that *P. vulgaris* originated in the Caucasus region and that its range expansion was accompanied by a reduction in genome-wide diversity.

Previous work proposed that the Carpathian and Alpine regions could have served as refugia for numerous plant species during more recent glaciations in the Quaternary (Trewick et al. 2002; Magri et al. 2006; Gömöry et al. 2003). Our demographic analyses suggest that, after a first population bottleneck likely associated with the initial range expansion ca. 105,000 to 201,000 ybp, population size quickly recovered and remained high for the past ca. 100,000 years in the Turkish population (Figs. 3a and 4). Following this initial genetic bottleneck, we identified a more recent genetic bottleneck in both the Slovakian (SK-D) and Swiss (CH-D) populations of *P. vulgaris* ca. 50,000 to 64,000 ybp (Fig.Figure 3aA and 4). This second bottleneck likely resulted from a subsequent population contraction caused by glacial expansion, suggesting that *P. vulgaris* could have survived in the Carpathian and Alpine regions during Quaternary glacial maxima (Bartha et al. 2015; Volkova et al. 2020). Although the observed genetic bottleneck is consistent with the role of the Carpathian and Alpine regions as refugia during the Quaternary glaciations, the sparsity of our population sampling prevents us from adequately testing this prediction. However, a recent demographic analysis that included a more comprehensive sampling of Central Europe populations (including Carpathian and Alpine populations) found genetic bottlenecks, thus supporting that these regions acted as cryptic refugia (Triest et al. 2024). The estimates of the genetic bottlenecks showed wide confidence intervals (Triest et al. 2024) that overlap with our estimates, thus supporting the fast range expansion observed in *P. vulgaris*. Overall, our results highlight the role of Quaternary glaciations in shaping the distributional range and genetic diversity of *P. vulgaris*, underscoring the importance of the Caucasian, Carpathian, and Alpine mountains as primary (Caucasus) and secondary (Carpathians and Alps) refugia and centers of genetic diversity for this species.

### The Colonization of England Preceded the Shift to Homostyly in *P. vulgaris*

During the Last Glacial Maximum (ca. 22,000 ybp), an extensive ice sheet covered the British Isles (Hughes et al. 2016). Unaffected areas in southern England were likely tundra environments unsuitable for most temperate plant species. Glacial retreat in this region provided favorable conditions for the establishment of temperate plant species in southern England, a process facilitated by a land bridge connecting England to mainland Europe 23,000 and 7,500 ybp (Combe et al. 2016). In line with the hypothesis of a postglacial expansion into England, our genome-wide phylogenomic analysis supports a recent colonization of the British Isles (Figs. 2d and 4). The recency of this event is reflected in low genomic differentiation among the English populations (Fig. 2b and c), along with lack of phylogenetic resolution among populations in the English clade with short external branches (Fig. 2d). Demographic analyses inferred the split between the easternmost (EN1-D) and westernmost (EN6-M) English populations to ca. 24,000 ybp (Fig. 4). Considering that retreat of the British-Irish Ice Sheet started ca. 24,000 ybp (Hughes et al. 2016), we propose this time as the likely temporal start for the expansion of *P. vulgaris* into the British Isles. The above-proposed timing of the colonization of England is consistent with the timing of post-glacial colonization of the island by *Alnus*, *Betula*, *Fraxinus*, and *Ulmus* trees inferred from pollen isochrone maps (Birks 1989), all of which form woodland communities associated with *P. vulgaris* (Jacquemyn et al. 2009). Overall, these results imply that the colonization of England occurred relatively recently, likely as a single event following glacial retreat, suggesting that the westernmost populations of *P. vulgaris* in England are the most recently established.

The ability to self-fertilize is thought to play a crucial role in facilitating the colonization of new habitats, including islands (Baker 1955). For example, previous work found that self-fertilizing variants of the ancestrally heterostylous *Eichhornia paniculata* colonized the Caribbean islands from continental America ca. 125,000 ybp (Barrett and Husband 1990; Ness et al. 2010). Similarly, the self-fertilizing ability of *P. vulgaris* homostyles arising in Europe could have facilitated the colonization of England. Alternatively, homostyly may have originated more recently in England in response to local scarcity of pollinators or mates, for example caused by climatic changes or severe habitat fragmentation (Mora-Carrera et al. 2023). Our genome-wide phylogenetic analyses revealed that all English populations formed a monophyletic group, supporting a single colonization of England (Fig. 2d). Additionally, each trimorphic population was found to be monophyletic, indicating that homostyles in EN4-T and EN5-T are more closely related to the heterostyles within each population than to any other heterostyles from England or continental Europe, thus suggesting that homostyles originated locally in England. Moreover, the single homostylous monomorphic population, EN6-M, is embedded within the English clade, which means that it is more closely related to English than European populations (Fig. 2d). The results of the genome-wide analyses presented here are congruent with previously published DNA sequencing analyses of *CYP^T^*, the *S*-locus gene that controls the shift from heterostyly to homostyly in *Primula* (Huu et al. 2016; Potente et al. 2022). Specifically, Mora-Carrera et al. (2023, 2024) found that multiple mutations in *CYP^T^* arose independently in different English populations of *P. vulgaris*. Because, like the entire S-locus, *CYP^T^* is hemizygous, any potentially loss-of-function mutation in it is dominant, causing the elongation of the style in homostylous flowers. Therefore, all available evidence supports the conclusion that independent shifts to homostyly occurred locally in two trimorphic (EN4-T and EN5-T) and one monomorphic (EN6-M) English populations of *P. vulgaris*, after island colonization. Further supporting the hypothesis of local origins for the English homostyles of *P. vulgaris*, the inferred demographic history of *P. vulgaris* (Figs. 3a and 4) reveals a genetic contraction possibly associated with the shift to homostyly in the monomorphic population EN6-M following colonization of the island after glacial retreat. Estimates of the timing of this genetic contraction based on our single- and multipopulation demographic analyses, despite being slightly different, indicate that this event occurred ca. 100 to 2,500 ybp (Figs. 3a and 4). This genetic contraction is relatively recent when compared to intraspecific transitions to selfing inferred in different plant systems. For instance, independent transitions to self-fertilization in *Leavenworthia alabamica* were dated around 150,000 and 12,000 to 48,000 ybp (Busch et al. 2011). Thus, to our knowledge, our results represent the most recent mating system transition in flowering plants reported to date. Therefore, taken together, our findings support the hypothesis that homostyly evolved locally in England, thus following island colonization, rather than in Europe, preceding island colonization. Although less parsimonious, our results cannot exclude the possibility that homostyly originated outside of England and subsequently colonized the British Isles. Identification of homostylous populations in continental Europe, such as the one in the Netherlands (Barmentlo et al. 2018), and analyses of its *CYP^T^* haplotypes are essential to help elucidate this question.

Fitting predictions of the genomic selfing syndrome (Wright et al. 2008; Cutter 2019), we detected substantial increase of LD (Fig. 5c), reduction of genetic diversity (Fig. 5a), and reduced efficacy of purifying selection (see below) in the homostyles of EN6-M. The increase of LD, likely due to selfing, in the homostyles of the monomorphic EN6-M compared to heterostylous populations in England was stronger than the increase of LD caused by genetic bottlenecks during the range expansion of *P. vulgaris*. This is expected because full selfing (selfing rate = 1) should half heterozygosity each generation resulting in a reduction of N_e_ (Wright et al. 2008). The above-mentioned increase of LD in EN6-M is congruent with previously published estimates of increased selfing rate and inbreeding coefficient in EN6-M as compared to heterostylous populations of *P. vulgaris* in England (Mora-Carrera et al. 2023). However, homostyles and heterostyles in trimorphic populations (EN4-T and EN5-T) displayed similar levels of genetic diversity and LD (Fig. 5c), although homostyles from these trimorphic populations did harbor less genetic diversity than the heterostyles of dimorphic populations (Table 1). The above-mentioned similar levels of genetic diversity between homostyles and heterostyles of trimorphic populations are unsurprising, given that pollen transport and fertilization between homostyles and heterostyles likely occur in trimorphic populations (Crosby 1949; Mora-Carrera et al. 2023). Additionally, the mentioned similarity of genetic diversity between homostyles and heterostyles of trimorphic populations is consistent with population-level estimates of outcrossing rate previously estimated for the same trimorphic populations, which were intermediate between those of dimorphic and monomorphic populations (Mora-Carrera et al. 2023). Overall, the intermediate values of genetic diversity, LD, and outcrossing rates in trimorphic populations might suggest that, to some extent, homostyles outcross with heterostyles of the same population. Thus, homostyles of *P. vulgaris* in trimorphic populations may display a mixed mating system Similarly, populations of *Arabis alpina* where individuals exhibit mixed mating also displayed intermediate levels of genetic diversity and LD compared to fully outcrossing and selfing populations (Laenen et al. 2018). Finally, mixed mating, rather than selfing, was also reported in the homostylous *Primula halleri* (de Vos et al. 2018). In summary, our results provide support for the emergence of the genomic selfing syndrome during the early stages of the shift to homostyly in *P. vulgaris* and highlight the potential role of mixed mating in delaying and/or counteracting the consequences of selfing.

### Range Expansions and Shift to Homostyly Are Associated with Reduced Purifying Selection and Differences in Genomic TE Content

The progressive reduction of *N_e_* associated with range expansions and transitions to selfing should lead to a reduction in the efficacy of purifying selection resulting in the accumulation of deleterious mutations (Ellegren and Galtier 2016). Evidence of the increased accumulation of deleterious mutations after range expansion and transition to selfing has been documented in *A. alpina* (Laenen et al. 2018; Zeitler et al. 2022). In agreement with these results, genome-wide analyses in *P. vulgaris* confirmed that both range expansion and mating system transition are associated with a reduction in the efficacy of purifying selection. Specifically, the DFE analyses showed a significant increase in the proportion of nearly neutral nonsynonymous sites in response to range expansion and shift to homostyly (*N_e_s* = 0 to 1; Fig. 5b and supplementary fig. S4a, Supplementary Material online). Although the π_N_/π_S_ analysis supports the results of the DFE analysis, the effect of purifying selection estimated with the former statistic was modest when compared to the latter (Fig. 2 and supplementary fig. S3, Supplementary Material online). Nonequilibrium demographic processes could lead to an underestimation of the strength of purifying selection (Brandvain and Wright 2016), as could be the case for the modest increase of π_N_/π_S_ we observed in western populations of *P. vulgaris*. However, DFE-alpha, the software we used to infer a relaxation of purifying selection based on the DFE, controls for an instantaneous population size change and has been shown to be robust under substantially more complex demographic scenarios (Keightley and Eyre-Walker 2007). Thus, it seems unlikely that the signal of a reduced efficacy of purifying selection we detected with DFE-alpha is entirely a confounding signal of demography but instead reflects a true biological consequence of range expansion and transition to homostyly. Thus, the present results are consistent with recent case studies where DFE and π_N_/π_S_ analyses inferred less efficient purifying selection in homostylous populations of the ancestrally heterostylous *Primula chungensis* (Wang et al. 2021) and *Primula oreodoxa* (Zeng et al. 2024), as well as in the homostylous *Linum trigynum* (Gutiérrez-Valencia et al. 2024). Overall, our results corroborate the conclusion that both range expansion and shift to homostyly are associated with reduced efficacy of purifying selection.

In addition to differences in the distribution of genetic diversity and efficacy of purifying selection, demographic processes such as genetic bottlenecks caused by range expansions can lead to both increases and decreases of TE genome content. The dynamics of expansion or contraction of TEs in the genome play an essential role in gene regulation, gene movement within the genome, pseudogenization, and the creation of new genes (Bennetzen and Wang 2014). Our results indicate that range expansions in *P. vulgaris* were indeed associated with changes in genomic TE content. However, the patterns of TE accumulation differed among TE superfamilies (Fig. 5d). Specifically, we found that western populations of *P. vulgaris* had a significantly higher genomic content of the *Mutator* TE superfamily than the easternmost population in Turkey (TR-D; Fig. 5d). *Mutator* TEs have high transposition rates and biased insertion close to coding regions, two features that make them highly mutagenic with potentially deleterious effects (Dupeyron et al. 2019). Therefore, it is likely that the increase of *Mutator* TE content in western populations reflects a relaxation in the efficacy of purifying selection due to a reduction of effective population size following range expansion, as proposed above. Similarly, we detected a higher genomic content of *Helitron* TE superfamily in SK-D and CH-D than in TR-D (supplementary fig. S5, Supplementary Material online). However, the same pattern was not found in the English populations, suggesting that the colonization of England could have purged some of these TEs (supplementary fig. S5, Supplementary Material online). In contrast to *Mutator* and *Helitron* TEs, genomic content of the *hAT* superfamily was higher in TR-D than in the remaining populations (Fig. 5d). This result could be explained either by an increase in the accumulation of *hAT* TEs in TR-D or a shrinkage of *hAT* TEs in the western populations (SK-D and CH-D) after the split between TR-D and the most western populations. The decrease of *hAT* TEs could have been facilitated by a purging effect possibly associated with genetic contractions following the out-of-Caucasus range expansion. In support of the latter explanation, previous theoretical work suggested that increased homozygosity after strong genetic contractions can lead to the purging of many recessive deleterious TEs (as those of the *hAT* superfamily) that are otherwise maintained in populations with high effective population size (Wright and Schoen 2000). Overall, our results suggest that similar demographic processes could cause an increase or decrease of TE genomic content, depending on differences of functional constraints among TE superfamilies.

Similarly to genetic bottlenecks resulting from range expansion, mating system transitions can also lead to changes in genomic TE content (Matilla et al. 2020). However, in contrast to our expectations, we found no differences of genomic TE content in any superfamily between dimorphic populations and any population where homostyly occurs (Fig. 5d). Therefore, our results indicate that the mating system transition had no effect on the genomic TE content of *P. vulgaris*. This result corroborates previous results that reported no differences in TE genomic content between the heterostylous *Linum tenue* and the homostylous *Linum trigyum* (Gutiérrez-Valencia et al. 2024). Previous theoretical work suggested that changes in TE genomic content caused by mating system transitions could take more than 2,000 generations to produce differences between outcrossers and selfers (Wright and Schoen 2000). Given that the origin of the fully homostylous EN6-M population of *P. vulgaris* is relatively recent (ca. 100 to 2,500 ybp; Figs. 3a and 4) and that the plant's life cycle is ca. 2 years, we estimate that ca. 50 to 1,250 generations have elapsed since the shift to homostyly in the monomorphic, monophyletic EN6-M (Fig. 2d). Therefore, we propose that, although other features of the genomic selfing syndrome, including reduced genetic diversity, increased LD, and lower efficacy of purifying selection have already evolved in homostylous plants of *P. vulgaris*, insufficient time has elapsed for detectable differences in the accumulation of TEs genomic content to occur. Taken together, our results failed to show greater accumulation of genomic TEs content as a consequence of the transition to selfing but did support the prediction that range expansions are associated with increased accumulation of TEs. However, the observed variation of TE genomic content among superfamilies suggests that the characteristics of each TE superfamily, such as insertion/excision rate and location of insertion, should cause differences in their deleteriousness, as suggested by Bonchev and Willi (2018). These differences should be considered in making predictions about how different demographic processes could affect the TE genomic content of different TE superfamilies in the genome.

## Conclusions

In this work, we performed the first study about the genome-wide consequences of both range expansion and recent shifts to homostyly in *P. vulgaris*. Our analyses, leveraging whole-genome resequencing data in combination with the high-quality reference genome of *P. veris*, allowed us to estimate, the timing of the out-of-Caucasus range expansion of *P. vulgaris*, providing new evidence that such seminal biogeographic event should be viewed in the context of the Quaternary glaciations. Furthermore, the present study provides the first temporal estimate for the intraspecific shift from heterostyly to homostyly in any *Primula* species. Despite the recency of this shift, we found evidence for the so-called genomic selfing syndrome in homostyles of *P. vulgaris*. These results provide a valuable framework for future studies aimed at determining whether the genomic consequences of the shift to homostyly are also accompanied by consequences at the phenotypic level, as reported in other plant mating system transitions. Finally, our work is the first one to date to characterize the changes of genomic TE content and composition stemming from both range expansion and shift to homostyly in any heterostylous/homostylous system, highlighting the role of functional constraints in the increase or decrease of TE genomic content among TE superfamilies.

## Materials and Methods

### Sampling and Sequencing of Plant Material

In spring of 2019, we collected and silica-gel dried leaf tissue from 105 individuals of *P. vulgaris* from six dimorphic, heterostylous populations (comprising only S- and L-morph individuals) in continental Europe (Turkey: TR-D; Slovakia: SK-D; Switzerland: CH-D) and England (EN1-D, EN2-D, and EN3-D); two trimorphic populations (comprising S-, L-, and H-morph individuals) in England (EN4-T and EN5-T); and one monomorphic population (comprising exclusively H-morph individuals) from England (EN6-M; Fig. 2a; Table 1). In total, we collected 74 heterostyles (37 L- and 37 S-individuals) and 31 homostyles. Total genomic DNA was extracted with the Maxwell extraction method (Promega, USA) at the Functional Genomics Center of the University of Zurich. Library preparation (300- to 400-bp fragment size) and paired-end sequencing (150 bp) were done by RAPiD GENOMICS (Gainesville, Florida) on an Illumina NovaSeq 6000 sequencer. In total, we obtained 7,077,866,510 paired-end sequencing reads with an average sequencing depth of 18.9 (± SD = 3.08).

### Preprocessing and SNP Filtering for Population Genetic Analyses

Illumina adapters were clipped from raw sequencing reads with Trimmonatic v0.38 (Bolger et al. 2014) in paired-end mode and with the option TruSeq3-PE.fa:5:10:20. Only read pairs that passed the trimming process were mapped against the *P. veris* chromosome-scale reference genome (Potente et al. 2022) using BWA-mem v7.17 (Li and Durbin 2009) with default options. Duplicated reads were marked with the MarkDuplicates tool in Picard v2.18.4 (http://broadinstitute.github.io/picard/). Calling of SNPs and insertion and deletion variants was done with HaplotypeCaller in the Genome Analysis Toolkit (GATK) v4.1.2.0 (McKenna et al. 2010) pipeline. The resulting GVCF files were combined and genotyped with the GATK tools CombineGVCFs and GenotypeGVCFs, respectively. Filtering of SNP variants was performed using the GATK tool SelectVariants with the following filters set: quality by depth > 2.0; mapping quality (MQ) > 40.0; strand bias < 60.0; MQ rank-sum test (MQRankSum) > −12.5; a rank-sum test (ReadPositionRankSum) > −8.0; and site read depth (DP < ½×) || (DP > 3×). Sites with fixed heterozygosity (i.e. InbreedingCoeff < −0.99) likely representing incorrect SNP calling (O’Leary et al. 2018; Pavan et al. 2020) were filtered out. Individual genotypes with allelic depth < 6 and >60 were set to no calls. Finally, sites with at least one individual deviating from allelic balance (AB) (i.e. 0.30 ≤ AB ≥ 0.70; where AB = alternative allele depth/total depth at the focal position), possibly causing erroneous heterozygous calls (Muyas et al. 2019), were filtered out with BCFtools v1.8 (Danecek et al. 2021). Only biallelic sites that mapped to the 11 chromosomes of the *P. veris* reference genome and with less than 20% of missing data were kept for all subsequent analyses.

### Genetic Structure and Phylogenetic Relationships

To describe the genetic structure of all sampled populations of *P. vulgaris*, we performed a PCA and applied a ML clustering method implemented in PLINK v1.9 (Purcell et al. 2007) and ADMIXTURE v1.3 (Alexander et al. 2009), respectively. For both analyses, genotype information from all 105 individuals was merged to a single VCF file using BCFtools v1.8 (Danecek et al. 2021). To avoid biases in both analyses, singletons were filtered out with VCFtools v0.1.17 (Danecek et al. 2011), and sites in LD (i.e. sites with R^2^ > 0.1 in nonoverlapping windows of 50,000 bp and steps of 10,000 bp) were pruned with PLINK v1.9. For the ADMIXTURE analysis, the number of subpopulations (*K*) ranged from 2 to 9, and the optimum was selected as the *K* value with the lowest cross-validation error. Results of the PCA and ADMIXTURE analyses were plotted in R v3.6.3 using custom scripts.

To determine phylogenetic relationships, we estimated a ML phylogeny based on 1,532,562 genome-wide SNPs using IQ-tree v2.1.2 (Minh et al. 2020) with 1,000 ultrafast bootstrap replicates (option -B 1000) and including ascertainment-bias correction for model finder (option -m MFP + ASC). We rooted the resulting tree with species from clades other than the *Primula* sect. *Primula* clade, namely *Primula boveana*, *P. halleri*, *Primula mistassinica*, *Primula nutans*, *Primula siamensis*, and *Primula verticillata*, following Stubbs et al. (2023) Read mapping and SNP calling for the outgroup specimens were performed as indicated above for the focal specimens.

### Demographic Inference

To determine whether range expansion and shift to homostyly in *P. vulgaris* were associated with genetic contractions and/or bottlenecks, we inferred single-population demographic histories using Stairway Plot v2 (https://github.com/xiaoming-liu/stairway-plot-v2; last accessed on January 30, 2022) with default options. We used four random break points ([nseq-2]/4, [nseq-2]/2, [nseq-2]*3/4, and nseq-2, where nseq = 2*[number of individuals analyzed]) as indicated in the manual. We specified a mutation rate of 1.23 × 10^−8^ per site per generation (Potente et al. 2022), adjusted from the mutation rate estimated for *P. veris* assuming a 2-year generation time (Boyd et al. 1990). To minimize the effect of selection on demographic inference, we retained only high-quality intergenic sites in the DNA data set used for all demographic analyses (Marchi et al. 2021). Specifically, using the *P. veris* reference genome and its annotation, we eliminated sites mapping to coding (including both introns and exons) regions and sites 2,000-bp upstream and downstream of each coding region. Moreover, we masked repetitive (including tandem repeats, target site duplications, and TEs) and putative centromeric regions based on the annotation of the *P. veris* reference genome (Potente et al. 2022). To ensure the use of an equal number of sites among populations and to reduce the effect of LD on demographic inference, we sampled 1 million random polymorphic sites in each population. The unfolded SFS of these neutral sites used as input to Stairway Plot v2 was estimated with easySFS (https://github.com/isaacovercast/easySFS; last accessed on February 20, 2022). For the unfolded SFS, we polarized the ancestral and derived state of each allele by assuming that alleles that matched the reference genome of *P. veris* represented the ancestral state.

Given that demographic histories inferred from single-population stairway plots can be unreliable due to their model-free nature (Lapierre et al. 2017), we used the results of the stairway plots results to create and subsequently test six different demographic models (supplementary fig. S1, Supplementary Material online) to the *P. vulgaris* data using fastmsimcoal2 v2.7 (Excoffier et al. 2021; see below for details of model selection). These six demographic models were informed by the results of the STRUCTURE, phylogenomic, and stairway plot analyses described above and aimed to identify the number and placement of bottlenecks putatively associated with the range expansion and shift to homostyly (see supplementary fig. S1, Supplementary Material online for model details). All six models included four populations (i.e. TR-D, heterostylous dimorphic population from Turkey; CH-D, heterostylous dimorphic population from Switzerland; EN1-D, heterostylous dimorphic population from England; and EN6-M, homostylous monomorphic population from England) that represent extremes of reproductive variation and distributional range of *P. vulgaris* as sampled for this study. We excluded SK-D because including these populations would have led to an increase in the number of demographic models to be tested, with the ensuing, substantial increase of computational time for the analyses. However, given the genetic similarity between SK-D and CH-D, we assume that using either one of these population should not considerably affect the conclusions of demographic analyses. Importantly in preliminary runs, we demonstrated that the bottleneck in TR-D predated the divergence between TR-D and all western populations (CH-D, EN1-D, and EN6-M; results not shown); thus, this bottleneck was no subsequently tested in the final six demographic models. Similar to the stairway plots analysis above, we retained only intergenic sites 2 kb away from coding regions that were present in all individuals (i.e. no missing data) were used for this analysis with *fastsimcoal2*. After these filters, we retained 27,541 polymorphic sites from the four populations for the analysis. The multidimensional SFS of these populations used as input for fastsimcoal2 was estimated with easySFS (https://github.com/isaacovercast/easySFS; last accessed on February 20, 2022) (supplementary fig. S6, Supplementary Material online). Parameter estimates of each model were obtained by choosing the fastsimcoal2 run that maximized the likelihood across 50 independent runs. We performed 40 expectation conditional-maximization cycles per run (-L), with 200,000 coalescent simulations per estimation (-n), a minimum site entry of ten (-C), and using the multidimensional SFS option (full command used: fsc2709 -t PREFIX.tpl -e PREFIX.est -d -n200000 -c32 -B32 -L40 -M --multiSFS -C 10 -x -q). We compared all models based on the AIC and the distribution of likelihoods from 100 simulated SFSs based on 10 million coalescent simulations under each model (-R 100 -n 10000000) (supplementary fig. S2, Supplementary Material online). We lumped together models with similar AIC for which the distribution of likelihoods overlapped, and then chose the (set of) model(s) in the lowest AIC category as the best model(s). We used parametric bootstraps to infer confidence intervals of the parameters of best-supported model(s). To do this, we generated 100 SFS based on the parameters of each model in the set of the best-supported model(s) as selected above. For each of these SFS, we reestimated the parameters by ML based on 20 independent runs per SFS, each based on 200,000 coalescent simulations per estimation (-n 200000) and 40 expectation conditional maximization cycles per run (-L 40) specifying initial values (--initvalues) of the respective best-supported model. From all these simulated parameters, we estimated confidence intervals by generating 40 SFS based on the population parameters that maximized the likelihood in the optimization procedure (*_maxL.par) using the -n40 -j -d -s0 -x -q -u commands. We then reestimated all population parameters using these 100 bootstrapped SFSs and estimated 95% confidence intervals from the distribution of simulated parameter values as the range between the empirical 2.5 and 97.5 percentiles.

### Genomic Consequences of the Range Expansion and Shift to Homostyly

#### Genome-Wide Diversity

To assess if the range expansion of *P. vulgaris* is associated with a reduction of genetic diversity, we tested whether a positive correlation exists between longitude and estimates of genome-wide diversity (i.e. east-to-west reduction of genetic diversity) using Spearman rank correlation test. To avoid conflating the effects of range expansion and mating system transitions, we focused exclusively on heterostylous dimorphic populations from Turkey to England (TR-D, SK-D, CH-D, EN1-D, EN2-D, and EN3-D).

To test if the shift from heterostyly to homostyly in *P. vulgaris* is associated with a reduction of genetic diversity, we compared estimates of genome-wide average genetic diversity between heterostylous and homostylous populations in England. For this analysis, we included EN1-D, EN2-D, and EN3-D as heterostylous populations and EN6-M as homostylous population. Additionally, we subdivided each trimorphic population into heterostyles (HE) and homostyles (HO); i.e. EN4-T was split into EN4-T-HE and EN4-T-HO and EN5-T into EN5-T-HE and EN5-T-HO. We focused exclusively on English populations here because we found that they stem from a single colonization of England (see Results; Fig. 2b and c). Thus, patterns of genetic diversity among these populations in England should primarily reflect the consequences of the mating system transition rather than range expansion. We used a Kruskal–Wallis test followed by a pairwise Wilcoxon rank test with Bonferroni correction to assess the evidence for differences in genome-wide diversity. All statistical analyses above were performed in Rv3.6.3 (R Core Team 2020).

We used Watterson's theta (θ_W_) and the pairwise nucleotide diversity (π) as estimates of genetic diversity. We calculated both estimates for synonymous (i.e. 4-fold degenerate) and nonsynonymous (i.e. 0-fold degenerate) sites. For this, we first annotated 4- and 0-fold degenerate sites using the script NewAnnotateRef.py (https://github.com/fabbyrob/science/tree/master/pileup_analyzers; last accessed on March 25, 2021) (Williamson et al. 2014). We then calculated per-site estimates of θ_W_ and π with scikit-allel v1.3.6 (Alistair et al. 2021) using nonoverlapping sliding windows of 50,000 sites. To avoid underestimation of π and θ_W_ caused by missing data, we used the accessibility mask featured in scikit-allel to correct for missing sites, as suggested by Korunes and Samuk (2021). Additionally, we estimated genome-wide Tajima's *D* with scikit-allel using nonoverlapping sliding windows of 50,000 sites.

Finally, to assess whether range expansion and shift to homostyly are each associated with increased LD, we estimated and compared the decay of LD among all populations. For this, we used popLDdecay (https://github.com/BGI-shenzhen/PopLDdecay; last accessed on April 12, 2021; Zhang et al. 2019) with default parameters except for the minor-allele frequency, which was modified in each population according to the sample size in order to exclude singletons.

### Strength of Purifying Selection

To evaluate the effects of range expansion and shift to homostyly on the efficacy of purifying selection in *P. vulgaris*, we estimated the ratio of nucleotide diversity at synonymous and nonsynonymous sites (π_N_/π_S_) and the DFE for all populations. To estimate π_N_/π_S_, we used the genome-wide diversity as indicated above, while to estimate the DFE of each population, we used DFE-alpha v2.15 (Keightley and Eyre-Walker 2007). Specifically, the DFE analysis estimates the proportion of nonsynonymous sites that are subject to increasing strengths of purifying selection as represented by *N_e_s*, where *N_e_* is the effective population size and *s* the selection coefficient against deleterious mutations. We divided the nonsynonymous sites based on the inferred value of *N_e_s*, as follows: nearly neutral sites (0 ≤ *N_e_s* < 1), weakly selected sites (1 ≤ *N_e_s* < 10), strongly selected sites (10 ≤ *N_e_s* < 100), and very strongly selected sites (100 ≤ *N_e_s* < ∞). This analysis is based on a comparison of the SFS at putatively neutral (synonymous) sites with the SFS at (nonsynonymous) sites putatively subject to natural selection. For the former SFS, we used intergenic sites located 2 kb away from coding regions, whereas for the latter SFS, we used 1 million 0-fold degenerate sites. Both SFS values were estimated as unfolded SFSs with easySFS (https://github.com/isaacovercast/easySFS; last accessed on February 20, 2021). To estimate 95% confidence intervals for *N_e_s*, we repeatedly applied the DFE analyses to 100 SFS generated by random sampling with replacement from the nonsynonymous SNPs.

To investigate whether range expansion reduced the efficacy of purifying selection, we tested whether longitude is negatively correlated with both π_N_/π_S_ and the proportion of nearly neutral sites (0 ≤ *N_e_s* < 1 category in the DFE) across all heterostylous dimorphic populations (TR-D, SK-D, CH-D, EN1-D, EN2-D, and EN3-D). Moreover, we expect a positive correlation between longitude and the proportion of weakly selected sites (1 ≤ *N_e_s* < 10) and strongly selected sites (10 ≤ *N_e_s* < 100). For these analyses, we applied a Spearman rank correlation test.

To investigate whether the shift to homostyly reduced the efficacy of purifying selection, we tested whether π_N_/π_S_ and the proportion of nearly neutral sites (0 ≤ *N_e_s* < 1 category in the DFE) is higher in homostylous (EN6-M, EN4-T-HO, and EN5-T-HO) than heterostylous (EN1-D, EN2-D, EN3-D, EN4-T-HE, and EN5-T-HE) populations in England. Moreover, we expect that heterostyles should have a higher proportion of weakly selected sites (1 ≤ *N_e_s* < 10) and strongly selected sites (10 ≤ *N_e_s* < 100) when compared to homostyles. For these analyses, we performed a Kruskal–Wallis test followed by a pairwise Wilcoxon rank test with Bonferroni correction.

### Genomic Content of TEs

We quantified the effects of range expansion and shift to homostyly on genomic TE content in *P. vulgaris* as follows. To determine whether range expansion is associated with changes in genomic TE content, we tested whether SK-D, CH-D, EN1-D, EN2-D, and EN3-D showed increased or decreased sequencing depth of TEs (used as a proxy for genomic TE content) relative to TR-D. To determine whether the shift to homostyly is associated with changes in genomic TE copy number, we tested whether homostyles in EN4-T, EN5T, and EN6-M showed increased or decreased TE sequencing depth relative to heterostyles in dimorphic and trimorphic populations in England (EN1-D, EN2-D, EN3-D, EN4-T, and EN5T).

Given that different TE superfamilies vary in the way they increase or decrease in frequency within the genome due to differences in replication mechanisms (Lee and Kim 2014), we analyzed the sequencing depth of the following TE superfamilies separately: *CACTA*, *hAT*, *Helitron*, *Mutator*, *PIF-Harbinger*, *Tc1-Mariner*, *LTR/Copia*, and *LTR/Gypsy*. Only TE superfamilies with at least 80% coverage (Wicker et al. 2007) were used for the analyses described below. We first determined the sequencing depth of different TE superfamilies by mapping the raw sequencing reads of each plant to the published TE library of the *P. veris* reference genome (Potente et al. 2022) using BWA-mem v7.17 (Li and Durbin 2009) with default options. Secondly, we used SAMtools v1.10 (Li et al. 2009) to estimate the sequencing depth of each TE family in each plant. Thirdly, to control for differences in sequencing depth among individuals, we normalized the TE sequencing depth of each individual by dividing the TE sequencing depth by the genome-wide sequencing depth of each individual. Finally, the TE genomic content of each population was obtained by averaging the normalized sequencing depth of all individuals belonging to the respective population. To test for the simultaneous interaction between population and TE superfamily on TE sequencing depth, we used generalized linear models of the type (TE sequencing depth) ∼ population * (TE superfamily) in Rv3.6.3 (R Core Team 2020), specifying a Gaussian distribution. For the analysis pertaining to range expansion, we specified the TE sequencing depth of TR-D as the intercept, whereas for the analysis regarding the consequences of shifts to homostyly, we specified EN1-D as the intercept using the “relevel” function on R.

## Supplementary Material

evae208_Supplementary_Data

## Data Availability

The 105 whole-genome sequences used in this study are deposited in SRA (BioProject ID: PRJNA1066534). Code and R scripts used to run the programs and create figures are available in (https://github.com/EmilianoMora/mora_carrera_etal_2024_GBE).

## References

[evae208-B1] Alexander DH , Novembre J, Lange K. Fast model-based estimation of ancestry in unrelated individuals. Genome Res. 2009:19(9):1655–1664. 10.1101/gr.094052.109. 19648217 PMC2752134

[evae208-B2] Alistair M , Murillo R, Ralph P, Kelleher J, Schelker M, Pisupati R, Rae S, Millar T. scikit-allel: v1.3.6. 2021. 10.5281/zenodo.13772087.

[evae208-B3] Arunkumar R , Ness RW, Wright SI, Barrett SCH. The evolution of selfing is accompanied by reduced efficacy of selection and purging of deleterious mutations. Genetics. 2015:199(3):817–829. 10.1534/genetics.114.172809. 25552275 PMC4349074

[evae208-B4] Baker HG . Self-compatibility and establishment after ‘'long-distance’ dispersal. Evolution. 1955:9:347–349. 10.2307/2405656.

[evae208-B5] Barmentlo SH , Meirmans PG, Luijten SH, Triest L, Oostermeijer JGB. Outbreeding depression and breeding system evolution in small, remnant populations of *Primula vulgaris*: consequences for genetic rescue. Conserv Genet. 2018:19(3):545–554. 10.1007/s10592-017-1031-x. 31007635 PMC6448329

[evae208-B6] Barrett SCH , Husband BC. Variation in outcrossing rates in *Eichhornia paniculata*: the role of demographic and reproductive factors. Plant Species Biol. 1990:5(1):41–55. 10.1111/j.1442-1984.1990.tb00191.x.

[evae208-B7] Bartha L , Sramkó G, Volkova PA, Surina B, Ivanov AL, Banciu HL. Patterns of plastid DNA differentiation in *Erythronium* (Liliaceae) are consistent with allopatric lineage divergence in Europe across longitude and latitude. Plant Syst Evol. 2015:301(6):1747–1758. 10.1007/s00606-014-1190-x.

[evae208-B8] Belaoussoff S , Shore JS. Floral correlates and fitness consequences of mating-system variation in *Turnera ulmifolia*. Evolution. 1995:49(3):545–556. 10.2307/2410278. 28565088

[evae208-B9] Bennetzen JL , Wang H. The contributions of transposable elements to the structure, function, and evolution of plant genomes. Ann Rev Plant Biol. 2014:65:505–530. 10.1146/annurev-arplant-050213-035811 24579996

[evae208-B10] Birks HJB . Holocene isochrone maps and patterns of tree-spreading in the British Isles. J Biogeogr. 1989:16(6):503–540. 10.2307/2845208.

[evae208-B11] Bolger AM , Lohse M, Usadel B. Trimmomatic: a flexible trimmer for Illumina sequence data. Bioinformatics. 2014:30:2114–2120. 10.1093/bioinformatics/btu170. 24695404 PMC4103590

[evae208-B12] Bonchev G , Willi Y. Accumulation of transposable elements in selfing populations of *Arabidopsis lyrata* supports the ectopic recombination model of transposon evolution. New Phytol. 2018:219(2):767–778. 10.1111/nph.15201. 29757461

[evae208-B13] Boutin TS , Le Rouzic A, Capy P. How does selfing affect the dynamics of selfish transposable elements? Mob DNA. 2012:3(1):5. 10.1186/1759-8753-3-5. 22394388 PMC3395816

[evae208-B14] Boyd M , Silvertown J, Tucker C. The ecology of homostyle and heterostyle primroses *Primula vulgaris* Huds. J Ecol. 1990:79(3):799–813. 10.2307/2260900.

[evae208-B15] Brandvain Y , Wright SI. The limits of natural selection in a nonequilibrium world. Trends Genet. 2016:32(4):201–210. 10.1016/j.tig.2016.01.004. 26874998

[evae208-B16] Busch JW , Joly S, Schoen DJ. Demographic signatures accompanying the evolution of selfing in *Leavenworthia alabamica*. Mol Biol Evol. 2011:28(5):1717–1729. 10.1093/molbev/msq352. 21199892

[evae208-B17] Charlesworth B . Effective population size and patterns of molecular evolution and variation. Nat Rev Genet. 2009:10(3):195–205. 10.1038/nrg2526. 19204717

[evae208-B18] Charlesworth D . Primrose homostyles: a classic case of possible balancing selection revisited. Mol Ecol. 2023:32(1):30–32. 10.1111/mec.16746. 36271781 PMC10099570

[evae208-B19] Combe FJ , Ellis JS, Lloyd KL, Cain B, Wheater CP, Harris WE. After the ice age: the impact of post-glacial dispersal on the phylogeography of a small mammal, *Muscardinus avellanarius*. Front Ecol Evol. 2016:4:72. 10.3389/fevo.2016.00072.

[evae208-B20] Crosby JL . High proportions of homostyle plants in populations of *Primula vulgaris*. Nature. 1940:145(3678):672–673. 10.1038/145672c0.

[evae208-B21] Crosby JL . Selection of an unfavourable gene-complex. Evolution. 1949:3(3):212–230. 10.2307/2405559. 18138378

[evae208-B22] Crosby JL . Outcrossing on homostyle primroses. Hered. 1959:13(1):127–131. 10.1038/hdy.1959.9.

[evae208-B23] Curtis J , Curtis CF. Homostyle primroses re-visited. I. Variation in time and space. Hered. 1985:54(2):227–234. 10.1038/hdy.1985.30.

[evae208-B24] Cutter AD . Reproductive transitions in plants and animals: selfing syndrome, sexual selection and speciation. New Phytol. 2019:224(3):1080–1094. 10.1111/nph.16075. 31336389

[evae208-B25] Danecek P , Auton A, Abecasis G, Albers CA, Banks E, DePristo MA, Handsaker RE, Lunter G, Marth GT, Sherry ST, et al The variant call format and VCFtools. Bioinform. 2011:27(15):2156–2158. 10.1093/bioinformatics/btr330. PMC3137218 21653522

[evae208-B26] Danecek P , Bonfield JK, Liddle J, Marshall J, Ohan V, Pollard MO, Whitwham A, Keane T, McCarthy SA, Davies RM, et al Twelve years of SAMtools and BCFtools. GigaScience. 2021:10(2):giab008. 10.1093/gigascience/giab008. 33590861 PMC7931819

[evae208-B27] de Vos JM , Hughes CE, Schneeweiss GM, Moore BR, Conti E. Heterostyly accelerates diversification via reduced extinction in primroses. Proc Biol Sci. 2014:281(1784):20140075. 10.1098/rspb.2014.0075. 24759859 PMC4043087

[evae208-B28] de Vos JM , Keller B, Zhang L-R, Nowak MD, Conti E. Mixed mating in homostylous species: genetic and experimental evidence from an alpine plant with variable herkogamy, *Primula halleri*. Int J Plant Sci. 2018:179(2):87–99. 10.1086/695527.

[evae208-B29] Dupeyron M , Singh KS, Bass C, Hayward A. Evolution of *Mutator* transposable elements across eukaryotic diversity. Mob DNA. 2019:10(1):12. 10.1186/s13100-019-0153-8. 30988700 PMC6446971

[evae208-B30] Ellegren H , Galtier N. Determinants of genetic diversity. Nat Rev Genet. 2016:17(7):422–433. 10.1038/nrg.2016.58. 27265362

[evae208-B31] Excoffier L , Foll M, Petit RJ. Genetic consequences of range expansions. Annu Rev Ecol Evol Syst. 2009:40(1):481–501. 10.1146/annurev.ecolsys.39.110707.173414.

[evae208-B32] Excoffier L , Marchi N, Marques DA, Matthey-Doret R, Gouy A, Sousa VC. *Fastsimcoal2*: demographic inference under complex evolutionary scenarios. Bioinformatics. 2021:37(24):4882–4885. 10.1093/bioinformatics/btab468. 34164653 PMC8665742

[evae208-B33] Goldberg EE , Kohn JR, Lande R, Robertson KA, Smith SA, Igić B. Species selection maintains self-incompatibility. Science. 2010:330(6003):493–495. 10.1126/science.1194513. 20966249

[evae208-B34] Gömöry D , Paule L, Shvadchak IM, Popescu F, Sulkowska M, Hynek V, Longauer R. Spatial patterns of the genetic differentiation in European Beech (*Fagus sylvatica* L.) at allozyme loci in the Carpathians and the adjacent regions. Silvae Genet. 2003:82:78–83.

[evae208-B35] Guggisberg A , Baroux C, Grossniklaus U, Conti E. Genomic origin and organization of the allopolyploid *Primula egaliksensis* investigated by in situ hybridization. Ann Bot. 2008:101(7):919–927. 10.1093/aob/mcn026. 18308718 PMC2710232

[evae208-B36] Guggisberg A , Mansion G, Kelso S, Conti E. Evolution of biogeographic patterns, ploidy levels, and breeding systems in a diploid–polyploid species complex of *Primula*. New Phytol. 2006:171(3):617–632. 10.1111/j.1469-8137.2006.01722.x. 16866963

[evae208-B37] Gutiérrez-Valencia J , Zervakis P-I, Postel Z, Fracassetti P, Losvik A, Mehrabi S, Bunikis I, Soler L, William Hughes P, Désamoré A, et al Genetic Causes and Genomic Consequences of Breakdown of Distyly in Linum trigynum. Molecular Biology and Evolution. 2024:41(5). 10.1093/molbev/msae087. PMC11114476 38709782

[evae208-B38] Hewitt GM . Genetic consequences of climatic oscillations in the Quaternary. Philos Trans R Soc Lond B Biol Sci. 2004:359(1442):183–195. 10.1098/rstb.2003.1388. 15101575 PMC1693318

[evae208-B39] Hughes PD , Gibbard PL, Ehlers J. Timing of glaciation during the last glacial cycle: evaluating the concept of a global ‘Last Glacial Maximum’ (LGM). Earth Sci Rev. 2013:125:171–198. 10.1016/j.earscirev.2013.07.003.

[evae208-B40] Hughes AL , Gyllencreutz R, Lohne ØS, Mangerud J, Svendsen JI. The last Eurasian ice sheets—a chronological database and time-slice reconstruction, DATED-1. Boreas. 2016:45(1):1–45. 10.1111/bor.12142.

[evae208-B41] Huu CN , Kappel C, Keller B, Sicard A, Takebayashi Y, Breuninger H, Nowak MD, Bäurle I, Himmelbach A, Burkart M, et al Presence versus absence of *CYP734A50* underlies the style-length dimorphism in primroses. eLife. 2016:5:e17956. 10.7554/eLife.17956. 27596932 PMC5012859

[evae208-B42] Jacquemyn H , Endels P, Brys R, Hermy M, Woodell SRJ. Biological Flora of the British Isles: *Primula vulgaris* Huds. *P. acaulis* (L.) Hill). J Ecol. 2009:97:812–833. 10.1111/j.1365-2745.2009.01513.x.

[evae208-B43] Jiang J , Xu YC, Zhang ZQ, Chen JF, Niu XM, Hou XH, Li XT, Wang L, Zhang YE, Ge S, et al Forces driving transposable element load variation during *Arabidopsis* range expansion. Plant Cell. 2022:36(4):840–862. 10.1093/plcell/koad296. PMC10980350 38036296

[evae208-B44] Johnston MO , Schoen DJ. Correlated evolution of self-fertilization and inbreeding depression: an experimental study of nine populations of *Amsinckia* (Boraginaceae). Evolution. 1996:50(4):1478–1491. 10.2307/2410885. 28565711

[evae208-B45] Keightley PD , Eyre-Walker A. Joint inference of the distribution of fitness effects of deleterious mutations and population demography based on nucleotide polymorphism frequencies. Genetics. 2007:177(4):2251–2261. 10.1534/genetics.107.080663. 18073430 PMC2219502

[evae208-B46] Keller B , Thomson JD, Conti E. Heterostyly promotes disassortative pollination and reduces sexual interference in Darwin's primroses: evidence from experimental studies. Funct Ecol. 2014:28(6):1413–1425. 10.1111/1365-2435.12274.

[evae208-B47] Korunes KL , Samuk K. Pixy: unbiased estimation of nucleotide diversity and divergence in the presence of missing data. Mol Ecol Res. 2021:21(4):1359–1368. 10.1111/1755-0998.13326. PMC8044049 33453139

[evae208-B48] Laenen B , Tedder A, Nowak MD, Toräng P, Wunder J, Wötzel S, Steige KA, Kourmpetis Y, Odong T, Drouzas AD, et al Demography and mating system shape the genome-wide impact of purifying selection in *Arabis alpina*. Proc Natl Acad Sci U S A. 2018:115(4):816–821. 10.1073/pnas.1707492115. 29301967 PMC5789905

[evae208-B49] Lapierre M , Lambert A, Achaz G. Accuracy of demographic inferences from the site frequency spectrum: the case of the Yoruba population. Genetics. 2017:206(1):439–449. 10.1534/genetics.116.192708. 28341655 PMC5419487

[evae208-B50] Lee S-I , Kim N-S. Transposable elements and genome size variations in plants. Genomics Inform. 2014:12:87. 25317107 10.5808/GI.2014.12.3.87 PMC4196380

[evae208-B51] Li J , Cocker JM, Wright J, Webster MA, McMullan M, Dyer S, Swarbreck D, Caccamo M, Oosterhout CV, Gilmartin PM. Genetic architecture and evolution of the S locus supergene in *Primula* vulgaris. Nat Plants. 2016:2(12):16188. 10.1038/nplants.2016.188. 27909301

[evae208-B52] Li H , Durbin R. Fast and accurate short read alignment with Burrows–Wheeler transform. Bioinformatics. 2009:25(14):1754–1760. 10.1093/bioinformatics/btp324. 19451168 PMC2705234

[evae208-B53] Li H , Handsaker B, Wysoker A, Fennell T, Ruan J, Homer N, Marth G, Abecasis G, Durbin R; 1000 Genome Project Data Processing Subgroup. The Sequence Alignment/Map format and SAMtools. Bioinform. 2009:25(16):2078–2079. 10.1093/bioinformatics/btp352. PMC2723002 19505943

[evae208-B54] Liu X , Fu Y-X. Stairway plot 2: demographic history inference with folded SNP frequency spectra. Genome Biol. 2020:21(1):280. 10.1186/s13059-020-02196-9. 33203475 PMC7670622

[evae208-B55] Lucek K , Willi Y. Drivers of linkage disequilibrium across a species’ geographic range. PLoS Genet. 2021:17(3):e1009477. 10.1371/journal.pgen.1009477. 33770075 PMC8026057

[evae208-B56] Magri D , Vendramin GG, Comps B, Dupanloup I, Geburek T, Gömöry D, Latałowa M, Litt T, Paule L, Roure JM, et al A new scenario for the Quaternary history of European beech populations: palaeobotanical evidence and genetic consequences. New Phytol. 2006:171(1):199–221. 10.1111/j.1469-8137.2006.01740.x. 16771995

[evae208-B57] Marchi N , Schlichta F, Excoffier L. Demographic inference. Curr Biol. 2021:31(6):R276–R279. 10.1016/j.cub.2021.01.053. 33756135

[evae208-B58] Mattila TM , Laenen B, Slotte T. Population genomics of transitions to selfing in Brassicaceae model systems. Methods in molecular biology. Statistical population genomics. New York, USA: Humana Press; 2020. p. 269–287. 10.1007/978-1-0716-0199-0_11 31975171

[evae208-B59] McKenna A , Hanna M, Banks E, Sivachenko A, Cibulskis K, Kernytsky A, Garimella K, Altshuler D, Gabriel S, Daly M, et al The Genome Analysis Toolkit: a MapReduce framework for analyzing next-generation DNA sequencing data. Genome Res. 2010:20(9):1297–1303. 10.1101/gr.107524.110. 20644199 PMC2928508

[evae208-B60] Minh BQ , Schmidt HA, Chernomor O, Schrempf D, Woodhams MD, Von Haeseler A, Lanfear R. IQ-TREE 2: new models and efficient methods for phylogenetic inference in the genomic era. Mol Biol Evol. 2020:37(5):1530–1534. 10.1093/molbev/msaa015. 32011700 PMC7182206

[evae208-B61] Mora-Carrera E , Stubbs RL, Keller B, Léveillé-Bourret É, de Vos JM, Szövényi P, Conti E. Different molecular changes underlie the same phenotypic transition: origins and consequences of independent shifts to homostyly within species. Mol Ecol. 2023:32(1):61–78. 10.1111/mec.16270. 34761469 PMC10078681

[evae208-B62] Mora-Carrera E , Stubbs RL, Potente G, Yousefi N, Keller B, De Vos JM, Szövényi P, Conti E. Genomic analyses elucidate S -locus evolution in response to intra-specific losses of distyly in *Primula vulgaris*. Ecol Evol. 2024:14:e10940. 10.1002/ece3.10940. 38516570 PMC10955462

[evae208-B63] Morgan MT . Transposable element number in mixed mating populations. Genet Res. 2001:77(03):261–275. 10.1017/S0016672301005067. 11486509

[evae208-B64] Muyas F , Bosio M, Puig A, Susak H, Domènech L, Escaramis G, Zapata L, Demidov G, Estivill X, Rabionet R, et al Allele balance bias identifies systematic genotyping errors and false disease associations. Hum Mutat. 2019:40(1):115–126. 10.1002/humu.23674. 30353964 PMC6587442

[evae208-B65] Ness RW , Wright SI, Barrett SCH. Mating-system variation, demographic history and patterns of nucleotide diversity in the tristylous plant *Eichhornia paniculata*. Genetics. 2010:184(2):381–392. 10.1534/genetics.109.110130. 19917767 PMC2828719

[evae208-B66] O’Leary SJ , Puritz JB, Willis SC, Hollenbeck CM, Portnoy DS. These aren’t the loci you’re looking for: principles of effective SNP filtering for molecular ecologists. Mol Ecol. 2018:27(16):3193–3206. 10.1111/mec.14792. 29987880

[evae208-B67] Pavan S , Delvento C, Ricciardi L, Lotti C, Ciani E, D'Agostino N. Recommendations for choosing the genotyping method and best practices for quality control in crop genome-wide association studies. Front Genet. 2020:11:447. 10.3389/fgene.2020.00447. 32587600 PMC7299185

[evae208-B68] Peischl S , Dupanloup I, Kirkpatrick M, Excoffier L. On the accumulation of deleterious mutations during range expansions. Mol Ecol. 2013:22(24):5972–5982. 10.1111/mec.12524. 24102784

[evae208-B69] Peischl S , Excoffier L. Expansion load: recessive mutations and the role of standing genetic variation. Mol Ecol. 2015:24(9):2084–2094. 10.1111/mec.13154. 25786336

[evae208-B70] Piper JG , Charlesworth B, Charlesworth D. Breeding system evolution in *Primula vulgaris* and the role of reproductive assurance. Hered. 1986:56(2):207–217. 10.1038/hdy.1986.33.

[evae208-B71] Potente G , Léveillé-Bourret É, Yousefi N, Choudhury RR, Keller B, Diop SI, Duijsings D, Pirovano W, Lenhard M, Szövényi P, et al Comparative genomics elucidates the origin of a supergene controlling floral heteromorphism. Mol Biol Evol. 2022:39(2):msac035. 10.1093/molbev/msac035. 35143659 PMC8859637

[evae208-B72] Purcell S , Neale B, Todd-Brown K, Thomas L, Ferreira MA, Bender D, Maller J, Sklar P, de Bakker PI, Daly MJ, et al PLINK: a tool set for whole-genome association and population-based linkage analyses. Am J Hum Genet. 2007:81(3):559–575. 10.1086/519795. 17701901 PMC1950838

[evae208-B73] Ren G , Mateo RG, Liu J, Suchan T, Alvarez N, Guisan A, Conti E, Salamin N. Genetic consequences of Quaternary climatic oscillations in the Himalayas: *Primula tibetica* as a case study based on restriction site-associated DNA sequencing. New Phytol. 2017:213(3):1500–1512. 10.1111/nph.14221. 27696413

[evae208-B74] Richards J . *Primula*. Portland, USA: Timber Press; 2003.

[evae208-B75] Rouzic AL , Deceliere G. Models of the population genetics of transposable elements. Genet Res. 2005:85(3):171–181. 10.1017/S0016672305007585. 16174335

[evae208-B76] Stubbs RL , Theodoridis S, Mora-Carrera E, Keller B, Yousefi N, Potente G, Léveillé-Bourret É, Celep F, Kochjarová J, Tedoradze G, et al Whole-genome analyses disentangle reticulate evolution of primroses in a biodiversity hotspot. New Phytol. 2023:237(2):656–671. 10.1111/nph.18525. 36210520 PMC10099377

[evae208-B77] Theodoridis S , Patsiou TS, Randin C, Conti E. Forecasting range shifts of a cold-adapted species under climate change: are genomic and ecological diversity within species crucial for future resilience? Ecography. 2018:41(8):1357–1369. 10.1111/ecog.03346.

[evae208-B78] Theodoridis S , Randin C, Broennimann O, Patsiou T, Conti E. Divergent and narrower climatic niches characterize polyploid species of European primroses in *Primula* sect. *Aleuritia*. J Biogeogr. 2013:40(7):1278–1289. 10.1111/jbi.12085.

[evae208-B79] Theodoridis S , Randin C, Szövényi P, Boucher FC, Patsiou TS, Conti E. How do cold-adapted plants respond to climatic cycles? Interglacial expansion explains current distribution and genomic diversity in *Primula farinosa L*. Syst Biol. 2017:66(5):715–736. 10.1093/sysbio/syw114. 28334079

[evae208-B80] Trewick SA , Morgan-Richards M, Russell SJ, Henderson S, Rumsey FJ, Pintér I, Barrett JA, Gibby M, Vogel JC. Polyploidy, phylogeography and *Pleistocene refugia* of the rockfern *Asplenium ceterach*: evidence from chloroplast DNA. Mol Ecol. 2002:11(10):2003–2012. 10.1046/j.1365-294X.2002.01583.x. 12296944

[evae208-B81] Triest L , Van Rossum F, Sramkó G, Sierens T, Volkova P. Over the hills and far away: phylogeography and demographic migration history of a dispersal-restricted primrose (*Primula vulgaris*). Front Ecol Evol. 2024:12:1333726. 10.3389/fevo.2024.1333726.

[evae208-B82] Volkova P , Laczkó L, Demina O, Schanzer I, Sramkó G. Out of Colchis: the colonization of Europe by *Primula vulgaris* Huds (Primulaceae). Acta Soc Bot Pol. 2020:89. 10.5586/asbp.89313.

[evae208-B83] Wang XJ , Barrett SCH, Zhong L, Wu ZK, Li DZ, Wang H, Zhou W. The genomic selfing syndrome accompanies the evolutionary breakdown of heterostyly. Mol Biol Evol. 2021:38(1):168–180. 10.1093/molbev/msaa199. 32761213 PMC7782863

[evae208-B84] Wicker T , Sabot F, Hua-Van A, Bennetzen JL, Capy P, Chalhoub B, Flavell A, Leroy P, Morgante M, Panaud O, et al A unified classification system for eukaryotic transposable elements. Nat Rev Genet. 2007:8(12):973–982. 10.1038/nrg2165. 17984973

[evae208-B85] Williamson RJ , Josephs EB, Platts AE, Hazzouri KM, Haudry A, Blanchette M, Wright SI. Evidence for widespread positive and negative selection in coding and conserved noncoding regions of *Capsella grandiflora*. PLoS Genet. 2014:10(9):e1004622. 10.1371/journal.pgen.1004622. 25255320 PMC4178662

[evae208-B86] Wohlfarth B . 2013. A review of Early Weichselian climate (MIS 5d-a) in Europe (Publication No. TR-13-03). Swedish Nuclear Fuel and Waste Management Co. SKB.

[evae208-B87] Wright SI , Ness RW, Foxe JP, Barrett SCH. Genomic consequences of outcrossing and selfing in plants. Int J Plant Sci. 2008:169(1):105–118. 10.1086/523366.

[evae208-B88] Wright SI , Schoen DJ. Transposon dynamics and the breeding system. Genetica. 2000:107(1/3):139–148. 10.1023/A:1003953126700. 10952207

[evae208-B89] Yuan S , Barrett SCH, Duan T, Qian X, Shi M, Zhang D. Ecological correlates and genetic consequences of evolutionary transitions from distyly to homostyly. Ann Bot. 2017:120(5):775–789. 10.1093/aob/mcx098. 28961784 PMC5691548

[evae208-B90] Zeitler L , Parisod C, Gilbert KJ. Purging due to self-fertilization does not prevent accumulation of expansion load. PLoS Genet. 2022:19(9):e1010883. 10.1371/journal.pgen.1010883. PMC10501686 37656747

[evae208-B91] Zeng ZH , Zhong L, Sun HY, Wu ZK, Wang X, Wang H, Li DZ, Barrett SCH, Zhou W. Parallel evolution of morphological and genomic selfing syndromes accompany the breakdown of heterostyly. New Phytol. 2024:242(1):302–331. 10.1111/nph.19522. 38214455

[evae208-B92] Zhang C , Dong S-S, Xu J-Y, He W-M, Yang T-L. PopLDdecay: a fast and effective tool for linkage disequilibrium decay analysis based on variant call format files. Bioinform. 2019:35(10):1786–1788. 10.1093/bioinformatics/bty875. 30321304

[evae208-B93] Zhong L , Barrett SCH, Wang XJ, Wu ZK, Sun HY, Li DZ, Wang H, Zhou W. Phylogenomic analysis reveals multiple evolutionary origins of selfing from outcrossing in a lineage of heterostylous plants. New Phytol. 2019:224(3):1290–1303. 10.1111/nph.15905. 31077611

[evae208-B94] Zhou W , Barrett SC, Li HD, Wu ZK, Wang XJ, Wang H, Li DZ. Phylogeographic insights on the evolutionary breakdown of heterostyly. New Phytol. 2017:214(3):1368–1380 10.1111/nph.14453. 28176339

