# Supplementary material for: Unveiling the Genome-Wide Consequences of Range Expansion and Mating System Transitions in Primula vulgaris
Source: Genome Biol Evol. 2024 Sep 28;16(10):evae208. doi: 10.1093/gbe/evae208 (PMC11469071; doi:10.1093/gbe/evae208)
Supplement: evae208_Supplementary_Data [file evae208_supplementary_data.docx]

**SUPPLEMENTARY MATERIAL**


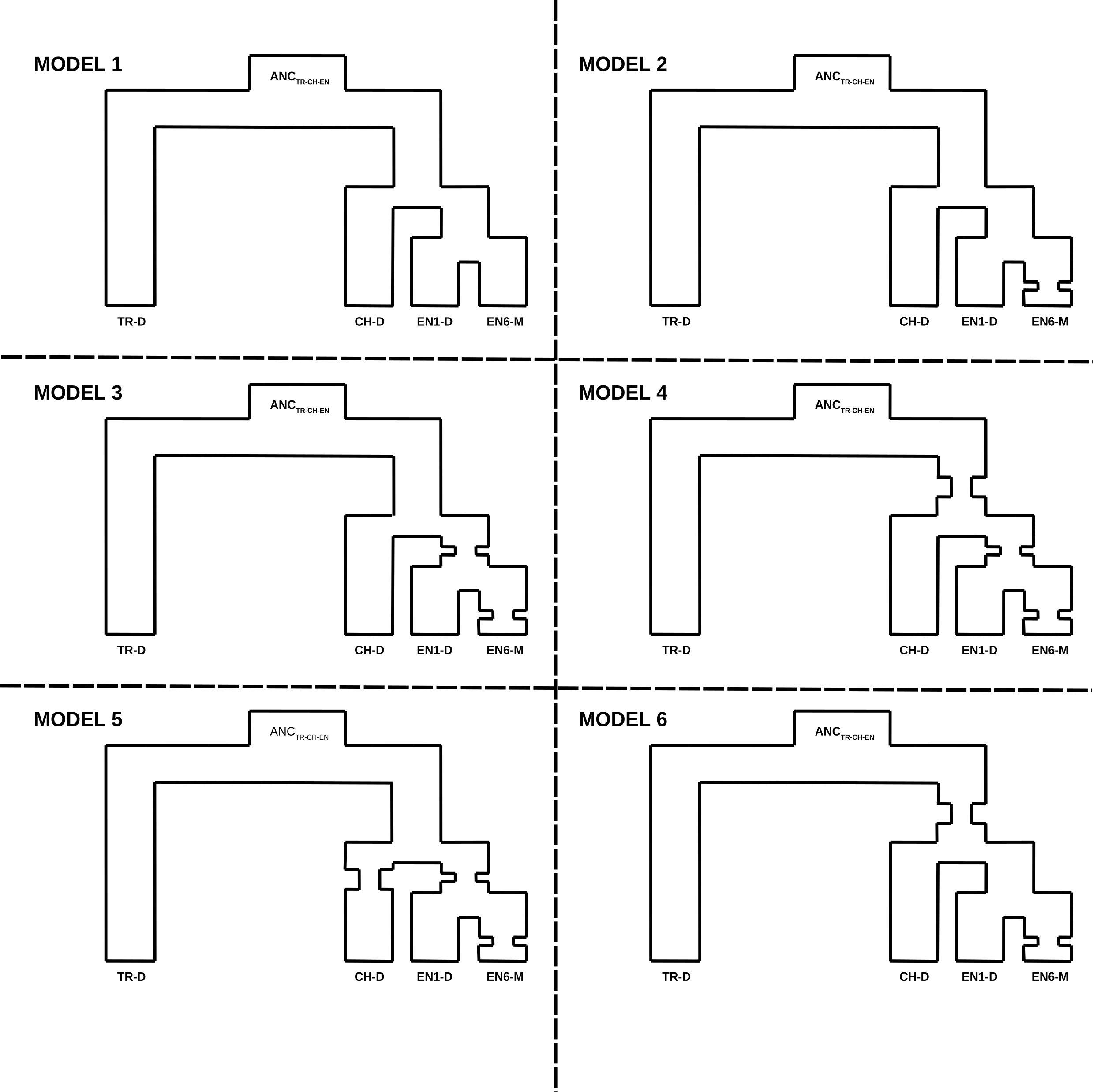


**Figure S1.** Demographic models of population divergence and genetic bottlenecks in *P. vulgaris* compared with *fastsimcoal2* (see Methods). The population-tree topology is the same for all models and reflects the results of the Maximum Likelihood (ML) phylogeny (Figure 2A). Models differ in the number and placement of population bottlenecks, and overall reflect the variation in single-population trajectories of effective population size in Figure 3. Model 1 assumes no bottleneck. Model 2 assumes one bottleneck associated with the fixation of homostyly in EN6-M. Model 3 assumes two bottlenecks associated with the colonization of England and the fixation of homostyly in EN6-M, respectively. Model 4 assumes three bottlenecks: two are the same as those in Model 3, and the third one is associated with the range expansion out of Turkey. Model 5 assumes three bottlenecks: two are the same as those in Model 3 and the third one is associated with the colonization of Central Europe. Model 6 assumes two bottlenecks, one associated with the fixation of homostyly in EN6-M (as in Model 1) and one associated with the range expansion out of Turkey. Preliminary runs in *fastsimcoal2* discarded a model in which the genetic bottleneck in TR-D shown in Figure 2A occurred before the split (results not shown). Hence, this bottleneck was not included in the models analyzed. The six models presented here were compared based on the multidimensional site frequency spectrum (SFS) and using three dimorphic populations from Turkey, Switzerland, and England (TR-D, CH-D, and EN1-D, respectively) and the homostylous monomorphic population (EN6-D). The validity of each model was assessed by visual inspection of the output of the par file interpreter script accompanying *fastsimcoal2* (ParFileViewer.r; <http://cmpg.unibe.ch/software/fastsimcoal27/R/ParFileViewer.r>).


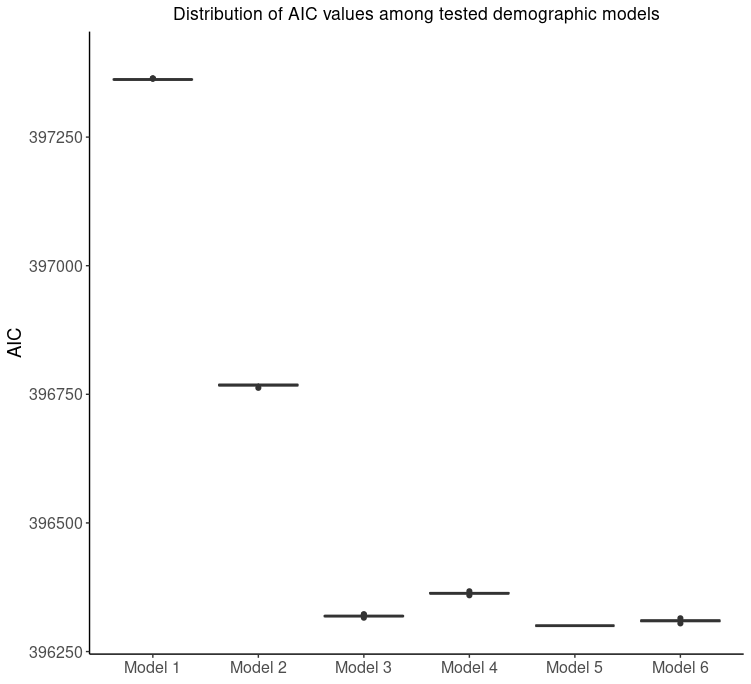


**Figure S2.** Boxplot showing the distribution of the Akaike Information Criterion (AIC) for all demographic models compared with *fastsimcoal2* (Excoffier et al., 2021). AIC values for each model were calculated based on the likelihood values obtained from 100 simulated site frequency spectra (SFSs), with 10 million coalescent simulations per simulated SFS (see Methods).


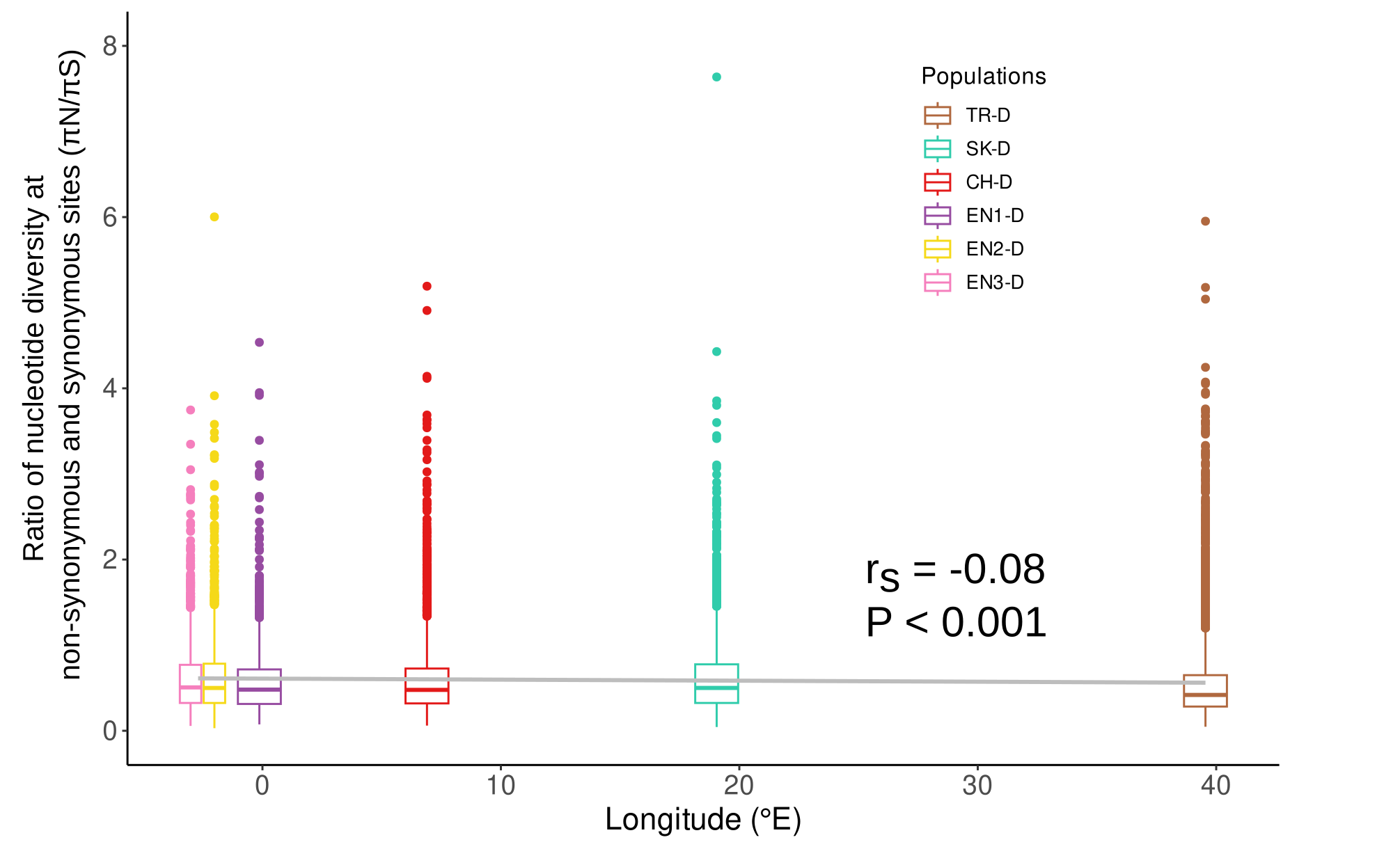


**Figure S3.** Reduction in the efficacy of purifying selection after the range expansion in *P. vulgaris*. Correlation plot showing the relationship between the ratio of nucleotide diversity at non-synonymous vs. synonymous sites (π_N_/π_S_) and longitude coordinates (°E). The significance of the correlation was assessed based on Spearman’s rank correlation coefficient (*r*_s_). Grey line indicates best linear fit.


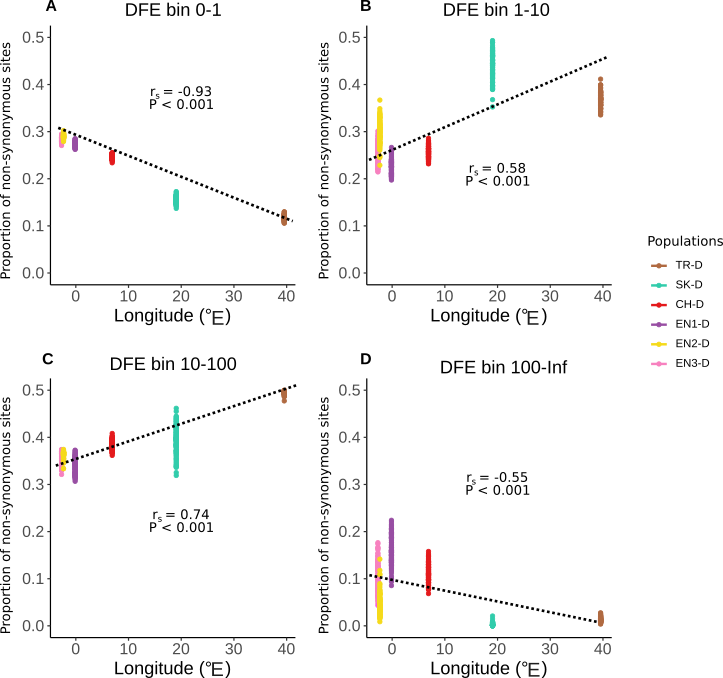


**Figure S4.** The effect of longitude on the distribution of fitness effects (DFE) among populations of *P. vulgaris*. Plots show the correlation between longitude coordinates (°E; x-axis) and the proportion of non-synonymous sites (y-axis) at increasing strength of purifying selection as follows: **A)** 0 ≤ *N_e_s* < 1; **B)** 1 ≤ *N_e_s* < 10; **C)** 10 ≤ *N_e_s* < 100; **D)** *N_e_s* ≥ 100. Significance of the correlation in each plot was estimated based on Spearman’s rank correlation coefficient (*r*_s_). The plot only includes the dimorphic populations (i.e., with S- and L-individuals) so that the effect of the range expansion may not be confounded by an effect of a transition from distyly to monostyly (see *Methods* for details). Doted line in all plots indicate best linear fit.


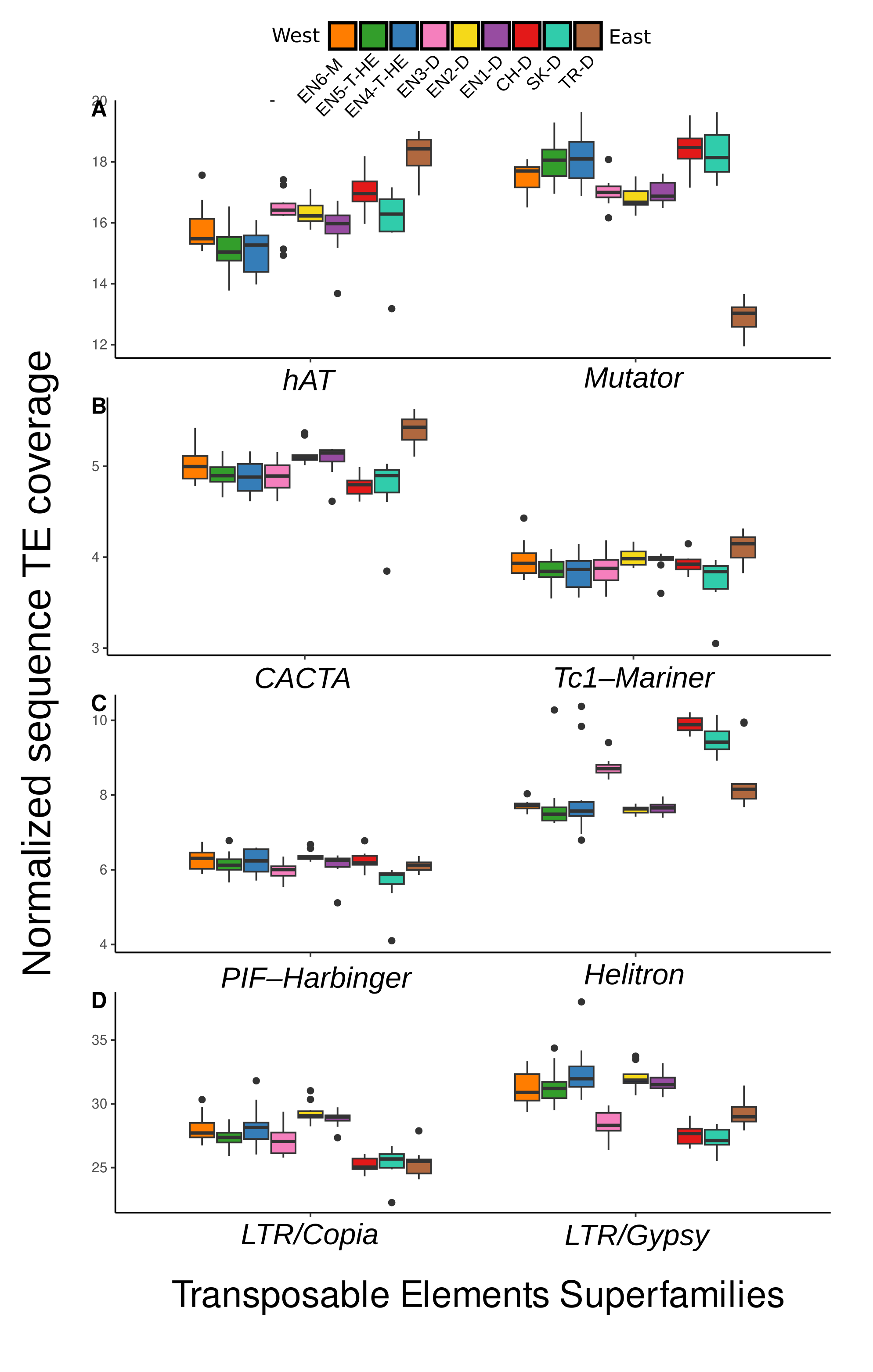


**Figure S5.** Accumulation of different TE superfamilies along the genome in nine populations of *P. vulgaris*. Boxplot showing the normalized sequencing depth of different TE superfamilies, including *CACTA*, *hAT*, *Helitron*, *Mutator*, *PIF*-*Harbinger*, *Tc1*-*Mariner*, *LTR*/*Copia*, and *LTR*/*Gypsy*. The TE sequencing depth was estimated as the mapping depth to each TE family, normalized by the sequencing sequencing depth of each sample (see Methods).


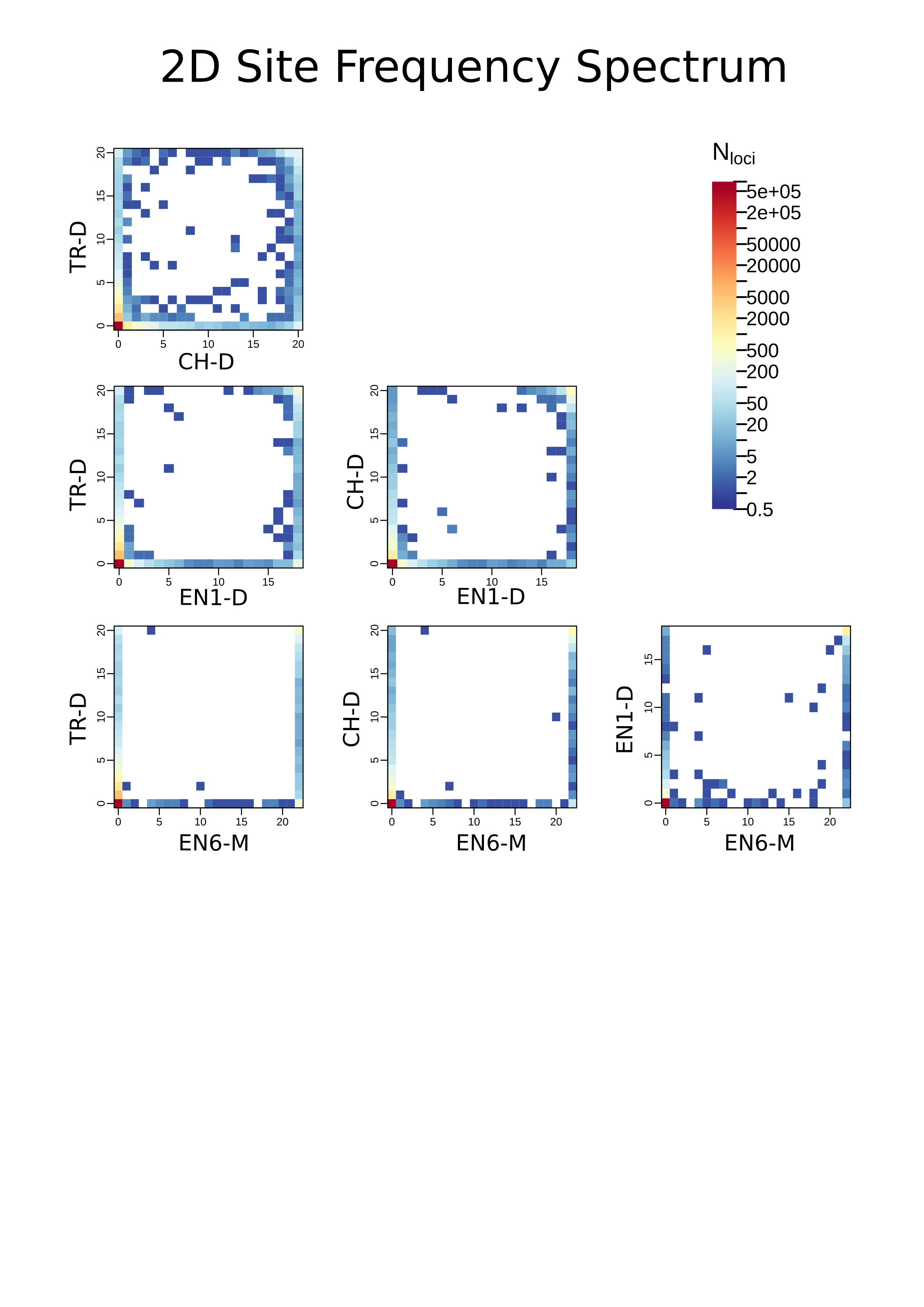


**Figure S6.** 2D unfolded site frequency spectrum (SFS) for all pairwise comparisons among the populations used for the demographic analysis with *fastsimcoal2* (Excoffier et al., 2021). For this analysis, three dimorphic populations from Turkey, Switzerland, and England (TR-D, CH-D, and EN1-D, respectively) and the homostylous monomorphic population in England (EN6-D) were used. The multiSFS was based on 23,804 biallelic, polymorphic, intergenic and repetitive-masked SNPs (see *Methods* for details).

**Table S1.** Cross-validation values for each number of subpopulations (*K*) in the STRUCTURE analysis analyses. See *Methods*. The *K* with the lowest cross-validation values is boldfaced.

**Table S2.** Reduction of genetic diversity associated with the transition from heterostyly to homostyly in *Primula vulgaris*. The table show P-values of the pairwise comparisons of **A)** nucleotide diversity (π) and **B)** Watterson’s theta (*θ*_w_) at 4-fold degenerate sites among all dimorphic (EN1-D, EN2-D, and EN3-D), trimorphic (EN4-T and EN5-T), and monomorphic (EN6-M) populations in England. Analyses were performed using pairwise Wilcoxon rank sum tests with Bonferroni corrections. See *Methods*.

**Table S3.** Results of the generalized linear model (glm) analyzing the effect of the range expansion in *P. vulgaris* on the genomic TE composition. For this analysis, normalized sequencing depth of each TE superfamily (*CACTA, hAT, Helitron, Mutator, PIF-Harbinger, Tc1-Mariner, LTR/Copia,* and *LTR/Gypsy*) was used as a proxy for genomic TE composition in each population. The value in the Turkish population (TR-D) was used as the intercept. See *Methods* for details.

**Table S4.-** Results of the generalized linear model (glm) analyzing the effect of the transition from heterostyly to homostyly in *P. vulgaris* on the genomic TE composition. For this analysis, normalized sequencing depth of each TE superfamily (*CACTA, hAT, Helitron, Mutator, PIF-Harbinger, Tc1-Mariner, LTR/Copia,* and *LTR/Gypsy*) was used as a proxy for genomic TE composition in each population. The value in the EN1-D population was used as the intercept. See *Methods* for details.
